# Human neuron chimeric mice reveal impairment of DVL-1-mediated neuronal migration by sevoflurane and potential treatment by rTMS

**DOI:** 10.1038/s12276-025-01425-0

**Published:** 2025-04-01

**Authors:** Youyi Zhao, Ya Zhao, Lirong Liang, Andi Chen, Yuqian Li, Ke Liu, Rougang Xie, Honghui Mao, Boyang Ren, Bosong Huang, Changhong Shi, Zhicheng Shao, Shengxi Wu, Yazhou Wang, Hui Zhang

**Affiliations:** 1 State Key Laboratory of Oral and Maxillofacial Reconstruction and Regeneration and National Clinical Research Center for Oral Diseases and Shaanxi Engineering Research, Center for Dental Materials and Advanced Manufacture, Department of Anesthesiology, School of Stomatology, Fourth Military Medical University, Xi’an, P. R. China; 2 Department of Neurobiology and Institute of Neurosciences, School of Basic Medicine, Fourth Military Medical University, Xi’an, P. R. China; 3 Laboratory Animal Center, Fourth Military Medical University, Xi’an, P. R. China; 4 Department of Neurology, Zhongshan Hospital, Institute for Translational Brain Research, State Key Laboratory of Medical Neurobiology and MOE Frontiers Center for Brain Science, Fudan University, Shanghai, P. R. China

**Keywords:** Cellular neuroscience, Embryonic stem cells, Neural progenitors, Cell culture, Social neuroscience

## Abstract

Whether early exposure to general anesthetics hurts human brain development is still under discussion. Animal studies have documented multiple neurotoxicities of repeated/prolonged exposure to sevoflurane (Sev, a commonly used pediatric anesthetic) at the neonatal stage. Its effects on human neural development remain elusive. Here, by investigating neural progenitor cells derived from two human embryonic stem cell lines, human cerebral organoids and human neuronal chimeric mice, we found that, although Sev inhibits neuronal differentiation and synaptogenesis of human neural progenitor cells in vitro, it only inhibits human neuronal migration in vivo. Chemogenetic activation of human neurons rescued the defects of cell migration and social dysfunction of Sev-pretreated human neuronal chimeric mice. Mechanistically, Sev inhibits DVL-1/Ca^2+^ signaling and multiple cell migration-related genes. Overexpressing DVL-1 enhanced the Ca^2+^ response, neuronal migration and social function of Sev-pretreated chimeric mice. Furthermore, specific modulation of human neurons by high-frequency transcranial magnetic stimulation not only activated DVL-1/Ca^2+^ signaling but also improved human neuronal migration and social function in chimeric mice. Our data demonstrate that early Sev exposure is toxic to human neuronal migration via inhibiting DVL-1 signaling and that transcranial magnetic stimulation could be potentially therapeutic.

## Early anesthesia impairs neuronal development and migration

Every year, millions of children and pregnant women undergo surgery with anesthesia, raising concerns about its safety. Some studies suggest that anesthesia, like sevoflurane (Sev), might harm brain development in young children, but results are mixed. Researchers explored Sev′s effects on human neurons using a new model involving human-mouse chimeric brains, which enabled them to observe the real-life effects of anesthesia. They exposed these models to Sev and found that while many toxic effects seen in lab settings were not present in living organisms, Sev did hinder human neuronal migration, leading to social behavior issues in human neuron chimeric mice. Further, they discovered that Sev affects a specific signaling pathway, which is crucial for neuron movement. By using transcranial magnetic stimulation (TMS), they could partially reverse these effects.

This summary was initially drafted using artificial intelligence, then revised and fact-checked by the author.

## Introduction

Each year, surgery and anesthesia are applied in approximately 2% of all pregnancies and over 6 million children around the world^1^. Concerns regarding the safety of applying general anesthetics to children or pregnant woman have been long discussed in the field. Animal studies have documented multiple neurotoxicities of repeated or prolonged exposure to general anesthesia at the neonatal stage^2,3^. However, clinical observations have produced inconsistent results^4^, with some researchers cautioning against the application of general anesthesia to children under the age of 3 years, while others think that anesthesia-related neurotoxicity is not a problem^5,6^.

Sevoflurane (Sev) is one of the most widely used pediatric anesthetics. Previous animal studies, including ours, have demonstrated that repeated neonatal exposure to Sev hurts cognitive function in the long term, possibly owing to neuronal loss, reactive oxygen species stress, neurogenesis impairment, synaptic deficits and even inflammation^7–12^. In the clinic, some researchers have reported that Sev induced neurodevelopmental disorder, while others found no significant effects of early Sev exposure on the long-term learning ability and intelligence quotient scores of children^13,14^. As most such clinical studies are retrospective and indirect^15,16^, it is important to elucidate the authentic effects of Sev by directly investigating its effects on human neurons.

Recent progress regarding human–mouse chimeric brain models offers a way of addressing this question in vivo^17^. In the present study, by adopting two human embryonic stem (ES) cell lines, human cerebral organoid (hCO) and human neuronal chimeric mice, we assessed the effects of Sev exposure on multiple aspects of human neuronal development. Our data showed that the in vivo microenvironment of chimeric mice erased most of the in vitro toxic effects of Sev, except for the inhibition of neuronal migration, which leads to social impairment of chimeric mice. Further, we demonstrated that this long-term toxic effect on neuronal migration is mediated by DVL-1 noncanonical Wnt signaling and that specifically modulating human neurons by transcranial magnetic stimulation (TMS) could partially rescue the defects.

## Methods

### Animals

All the NOD-SCID mice were bought from the animal center of the Fourth Military Medical University. Neonatal NOD-SCID mice (P0–P2) were used to make chimeric mice. All the chimeric mice included in the experiments were 2-month-old males. All animal experiments were carried out under protocols approved by the Animal Care and Use Committee of Fourth Military Medical University.

### Neural induction of hES cell culture and Sev treatment

The human embryonic stem (hES) cell line H8 (RRID: CVCL_B207) was obtained from Prof. Wei Jiang (Wuhan University), and cell line H9 (RRID: CVCL_9773) was bought from Nuwacell Biotechnology Co. Ltd. Cells were maintained in mTeSR1 feeder-free cell medium. Neural induction was conducted according to a previous protocol as reported^18^, with minor modifications. Briefly, hES cells were digested using accutase and cultured in suspension using neural induction medium for 7 days. After embryonic body (EB) formation, EBs were transferred to six-well plates precoated with Poly-D-lysine (PDL). Cells were cultured with neural induction medium and passaged every 6–7 days. Then, cells were seeded onto coverslips precoated with PDL and cultured with neuron-differentiation medium (neurobasal containing 2% B27, 10 ng ml^−1^ brain-derived neurotrophic factor and 10 ng ml^−1^ glial cell-derived neurotrophic factor).

For Sev exposure, 4.1% Sev (2 minimum alveolar concentration for human) was delivered from an anesthesia machine to a sealed plastic box in a 37 °C incubator as described previously^19,20^. An anesthetic gas monitor (Drager) was used to continuously monitor the concentrations of carbon dioxide, oxygen and Sev. Neural progenitor cells (NPCs) were treated with Sev (21% O_2_, 5% CO_2_ and 4.1% Sev, H20110142) for 6 h.

For cell migration, Sev treatment was performed after 23 days of neural induction. Then, human NPCs (hNPCs) were cultured in suspension for 7 days for sphere formation. Neural spheres were seeded onto PDL-precoated six-well plates, and cell migration was assessed 24 h later.

### hCO culture and Sev treatment

hCOs were cultured as described, with minor modifications^21^. hES cells were seeded in 96-well plate (9,000 cells per well) and cultured with EB medium (a 4:1 mixture of DF12 and Knockout Serum Replacement) containing 2 mM GlutaMAX, 0.1 mM non-essential amino acids (NEAA, 10 ng ml^−1^ Noggin and 1 mM SB431542) for 5 days. Then, organoids were transferred to low-attachment six-well plates and cultured with a 1:1 mixture of EB medium and NPC medium (DF12 containing 1% N2, 0.1 mM NEAA, 2 mM GlutaMAX, 1 mM CHIR 99021 and 1 mM SB431542) for 7 days. Then, organoids were cultured with NPC medium in rotation at 80 rpm. Three days later, organoids were cultured with mature medium (49% DF12, 49% Neurobasal, 1% N2, 1% B27, 2 mM GlutaMAX, 2.8 ng ml^−1^ insulin and 0.1 mM NEAA). Thirty days later, organoids were treated with 4.1% Sev for 6 h. Cell migration was assessed 5 days after Sev treatment.

### Humanized neuronal chimeric mice and Sev treatment

Humanized neuronal chimeric mice were established as described^22^. Briefly, hNPCs/human neurons (h-neurons) were digested and suspended in culture medium containing 5 mM EGTA, as suggested^23^. Then, 1–2 × 10^5^ cells were transplanted into the bilateral forebrain cortex of P0 neonatal NOD-SCID pups to make chimeric mouse. At 1 or 2 months following transplantation, mice were used for experiments. Two Sev exposure protocols were adopted: (1) hNPCs/h-neurons were treated with 4.1% Sev for 6 h and transplanted 24 h later to make chimeric mice. Chimeric mice made by control hNPCs/h-neurons were used as control or (2) from P6, chimeric mice were treated with 3% Sev (minimum alveolar concentration for mouse) +60% oxygen (balanced with nitrogen) for 2 h per day for 3 consecutive days as described previously^24,25^. In parallel, chimeric mice were treated with 60% oxygen (balanced with nitrogen) for 2 h per day for 3 consecutive days as a control. To ensure consistency in the data, all treatments were carried out at 8:00–10:00.

### Immunohisto(cyto)chemistry

For immunohistochemistry, mice were perfused intracardially with 4% paraformaldehyde phosphate buffer. Serial coronal sections were prepared and blocked by PBS containing 3% BSA and 0.3% Triton-X100, and then incubated with primary antibodies overnight at room temperature with the following: rabbit anti-OCT4 (GeneTex, GTX101497, RRID: AB_10618784, 1:200), rabbit anti-Sox2 (GeneTex, GTX101507, RRID: AB_2038021, 1:400), mouse anti-Nestin (GeneTex, GTX630201, RRID: AB_2888203, 1:200), rabbit anti-Pax6 (GeneTex, GTX113241, RRID: AB_1951119, 1:200), rabbit anti-Ki67 (GeneTex, GTX16667, RRID: AB_422351, 1:200), goat anti-GFP (GeneTex, GTX26673, RRID: AB_371426, 1:400), mouse anti-Tuj-1 (abcam, ab78078, RRID: AB_2256751, 1:100), rabbit anti-CaMKII (GeneTex, GTX135117, RRID: AB_2887447, 1:400), rabbit anti-VGLUT2 (CST, 71555, RRID: AB_2799805, 1:1000), guinea pig anti-DCX (Millipore, AB2253, RRID: AB_1586992, 1:500) and rat anti-BrdU (abcam, ab6326, RRID: AB_305426, 1:200). After washing with PBS, the corresponding secondary antibodies conjugated with donkey anti-rabbit (Alexa Fluor 594, Invitrogen, A-21207 RRID: AB_141637, 1:800), donkey anti-mouse (Alexa Fluor 594, Invitrogen, A-21203, RRID: AB_141633, 1:800), donkey anti-goat (Alexa Fluor 488, Invitrogen, A-11055, RRID: AB_2534102, 1:800), donkey anti-rat (Alexa Fluor 488, Invitrogen, A-21209, RRID: AB_2535795, 1:800) and donkey anti-guinea pig (Alexa Fluor 594, Jackson Immunoresearch, 706-585-148, RRID: AB_2340474, 1:500) were incubated with the sections for 2–4 h at room temperature protected from light. After washing with PBS, the sections were counterstained with Hoechst33342 (1:1,000, Sigma) for 20 min. All images were acquired by a confocal microscope (FV3000, Olympus). For quantification, ImageJ software was employed for the manual counting of single- or double-stained cells.

### Western blotting

Tissues or cells were homogenized in RIPA lysis buffer containing a proteinase inhibitor cocktail. Protein samples were separated by using a 10–15% gel. After SDS–PAGE, proteins were transferred to a PVDF membrane. Membranes were blocked with TBS containing 5% non-fat milk and 0.1% Tween20 and then incubated with primary antibodies overnight at 4 °C with the following: mouse anti-β-actin (proteintech, 66009-1, RRID: AB_2687938, 1:5,000), rabbit anti-PSD-95 (abcam, ab18258, RRID: AB_444362, 1:200), mouse anti-β-Tubulin III (abcam, ab78078, RRID: AB_2256751, 1:1,000), mouse anti-Wnt5a (abcam, ab229200, RRID: AB_2890100, 1:500), rabbit anti-DVL-1 (proteintch, 27384-1-AP, RRID: AB_2880859, 1:1,000), rabbit anti-CaMKII (Genetex, 135117, RRID: AB_2887447, 1:1,000), rabbit anti-Rho-A (abcam, ab187027, RRID: AB_2827434, 1:2,000), rabbit anti-β-catenin (Cell Signaling Technology, 8480s, RRID: AB_11127855, 1:900), rabbit anti-pGSK-3β (Cell Signaling Technology, 5558s, RRID: AB_10013750, 1:900), rabbit anti-Axin2 (abcam, ab32197, RRID: AB_2290204, 1:900), rabbit anti-DVL-1 (proteintch, 27384-1-AP, RRID: AB_2880859, 1:1,000), rabbit anti-SYP (abcam, ab32127, RRID: AB_2286949, 1:1,000) and mouse anti-RAC-1 (Proteintech, 66122-1-Ig, RRID: AB_2881521, 1:1,000). After washing with TBST, membranes were incubated with horseradish peroxidase-conjugated anti-rabbit and horseradish peroxidase-conjugated anti-mouse (1:5,000; Bioss, bs-0296G, RRID: AB_10856484, and worldbio, WH-002) for 1 h at room temperature. Bands were visualized with an ECL kit (Thermo, 32106). Images were analyzed by using ImageJ (RRID: SCR 003070).

### Real-time RT–PCR

RNA was extracted using Trizol reagent (Thermo Fisher Scientific, 15596018). cDNA was made by the PrimeScript RT Master Mix (TaKaRa, RR036A). PCR was performed using TB Green Premix Ex Taq II (TaKaRa, RR820). The ΔΔCt method was used for comparisons among different experimental groups. The primer sequences were as follows: *FGF17*: CTTGGCTTCTCTGGGACTCT, GGTCCCTCACGTACTGAGTTT; *LHX*: GCACACAGTCGCCCTCATA, AGTGAAGGTCAGCGAGAACG; *MIXL*: TTTTCTCCCCTCTTCCAGGTAT, GGGCAGGCAGTTCACATCTA; *Pitx2*: AAGGAAAGCTAACGCCGAC, AAGGAAAGCTAACGCCGAC; *STC1*: AAGATGGCGACCACCAAAGT, GCAGTGACGCTCATAAGGGA; *TEK*: GCGAGATGGATAGGGCTTGA, GCACAGAAGCAGGCTGTAAC; *DVL-1*: TCCTCACTAACCAGCTCCGT, GGCGCTCATGTCACTCTTCA; and *Wnt5a*: TCCTCTCGCCCATGGAATTA, CATTGCACTTCCAGCCATCC.

### Calcium imaging

The calcium response was measured as described previously^26^. Briefly, h-neurons were incubated with media containing 4 μM Rhod-4 AM (Rhod-4, AAT Bioquest Inc., 20551) for 15 min. After resting, Ca^2+^ levels were recorded and 3 mM KCl was added. Fluorescent signals were excited at 561 nm and imaged every 1 s for 180 s using a confocal microscope (Olympus, FV3000). Calcium influx and resting Ca^2+^ levels were measured by image analysis software Cellcens (Olympus). For each experimental condition, the Δ*F*/*F* value of more than 100 cells were caculated using Igor Pro software (WaveMetrics). The results from ≥3 independent experiments were averaged.

### Behavior assays

#### Three-chamber test

The three-chamber test was conducted as previously described^27^. Briefly, after habituation, a stimulus mouse was placed in the cylinder in the ‘social chamber’, with the cylinder in the ‘non-social chamber’ remaining empty. The time the test mice spent in the social versus non-social chambers was measured. The behavior of each mouse was video recorded. Each chamber was cleaned with 75% ethanol between tests. The behavior was analyzed using SMART3·0 software (Panlab Harvard Apparatus). The (time social − time non-social)/(time social + time non-social) was calculated as the preference score.

#### Resident–juvenile intruder test

The test was performed as previously described^27,28^. Briefly, the resident mouse (test mouse) was allowed to explore freely in his home cage. An intruder mouse (novel, 3–4 weeks old) was put into the resident cage. A juvenile intruder was used to avoid mutual aggression. The test mouse was allowed to explore the intruder mouse freely for 10 min. The time and frequency of direct contacts were measured.

#### Open field test

The open field test was carried out in a white opaque plastic chamber (50 × 50 × 35 cm^3^) as described previously^29^ with minor modification. The open field was divided into 25 squares with the same area. The central nine squares were defined as the central area, and the remaining as the periphery area. For each test, a mouse was gently placed in one corner, and the movement was recorded for 5 min with a video tracking system. The time spent and distance traveled in the central area and the total distance traveled in the field were measured using SMART software (SMART 3.0, Panlab S.L.U.). Between each test, 75% ethanol was used to clean the open field area.

#### Elevated cross maze test

The elevated plus maze test was performed as described previously^30^. The maze was placed 50 cm above the floor and consisted of two open arms and two closed arms (30 × 5 cm^2^ and 15 cm wall height for the closed arms). Each mouse was placed onto the center area, heading toward the same open arm and videotaped in the following 5 min. The time spent and the moving distance in the open arms, and the total movements in both open and closed arms were analyzed using the software SMART 3.0. The maze was cleaned by using 75% ethanol between tests.

#### Fear conditioned test

All mice were pre-exposed to the startle chambers (SanDiego Instruments) 3 days before training. During cued fear training, mice received five paired conditioned stimulus tones (30 s, 6 or 12 kHz, 90 db) and unconditioned stimulus shock (500 ms, 1.0 mA) trials with a 5 min interval. The startle response to the shocks and the percentage of time spent freezing to the tones were measured by using Xmaze software (XinRuan Informatics Co.).

#### Novel object exploration test

On day 1, mice were put into the open field (40 × 40 × 35 cm^3^) for 10 min of free exploration. On day 2, mice were first put into the same open field with two identical Lego blocks for 10 min exploration. One hour later, one Lego block was replaced with a novel block that had different shape, and the mice were put back for 5 min exploration. On day 3, the mice were tested exactly the same as day 2 except that the novel block was replaced by another one the mice never encountered before. The ratio of exploration time on novel object and old object was calculated.

### Patch-clamp recording

Transverse brain slices of control and Sev-pretreated chimeric mice (350–450 μm thickness) were freshly prepared and recovered in an incubation solution (95 mM NaCl, 1.8 mM KCl, 1.2 mM KH_2_PO_4_, 0.5 mM CaCl_2_, 7 mM MgSO_4_, 26 mM NaHCO_3_, 15 mM glucose and 50 mM sucrose, oxygenated with 95% O_2_, 5% CO_2_ at pH 7.4) at room temperature for 1 h before recording. For recording, a slice was transferred into the recording chamber, which was perfused with oxygenated recording solution (identical to the incubation solution except for the following components: 127 mM NaCl, 2.4 mM CaCl_2_, 1.3 mM MgSO_4_ and 0 mM sucrose). The slices were illuminated with a monochromator and visualized with an upright fluorescence microscope (BX51WI, Olympus), equipped with Dodt-infrared optics using a 40× 0.80 NA water immersion objective and a cooled CCD camera (TILL Photonics). Standard whole-cell patch-clamp recordings were performed with glass pipettes with a resistance of 4–6 MΩ on GFP-positive neurons. The pipette solution was made of 135 mM K-gluconate, 5 mM KCl, 0.5 mM CaCl_2_, 2 mM MgCl_2_, 5 mM EGTA, 5 mM HEPES and 5 mM Mg ATP (pH 7.4, osmolarity 300 mOsm). Recordings were acquired with an Axon700B amplifier (Molecular Devices Corporation) and Clampex 9.2 software. Signals were low-pass filtered at 5 kHz, sampled at 10 kHz and analyzed offline with Clampfit 10.6 software. The membrane potential was held at −70 mV for recording spontaneous excitatory postsynaptic currents.

### Chemogenetic manipulation

The hES cell line H8 was transfected with rLV-EF1a-hM3D(Gq)-mCherry-WPRE (LV-0910, 2.0 × 10^8^ TU ml^−1^, BrainVTA Co. Ltd.). Monoclonal mCherry-positive cells were expanded and induced toward neuronal fate. Subsequently, the cells were collected for transplantation. When chimeric mice were 2 months old, 1 mg kg^−1^ clozapine-*N*-oxide (CNO; 6329, Tocris Bioscience) was injected for 5 consecutive days. Behavior analysis was conducted 1 h after the last CNO injection.

### rTMS treatment

#### Brain region specificity test of precisely targeted TMS

A newly developed TMS device, equipped with a 13-layer three-turn ‘8’-shape coil that can focus the stimulation region within a minimal size of 0.5 mm^3^ (Black Dolphin IT-TMS, Solide Company), was adopted. Before the formal experiments, the brain region specificity was tested by stimulating the hindlimb region of the right motor cortex. Upon single TMS stimulation, movement of the bilateral hindlimbs was videoed. At the same time, electromyographic activity in the bilateral hindlimbs was recorded as described previously^31^. Briefly, the positive electrode was inserted into the front end of the gastrocnemius muscle. The negative electrode was inserted into the back end of the gastrocnemius muscle and the grounding electrode was inserted into the root of the mouse’s tail. The highest signal sampling rate was set at 32,000 Hz. The single-channel signal sampling rate was set at 8000 Hz. The signal was filtered through a 50 Hz band-stopping filter, a 20–480 Hz band-passing filter and a power frequency harmonic filter (Solide Company).

#### Precisely targeted rTMS treatment

Repeated TMS (rTMS) was carried out by placing the probe 1–2 mm above the transplantation region. The parameters of rTMS were as follows: 10 Hz, stimulating pulse intensity 40% of the maximum power of the rTMS device and 800 pulses per day (20 stimulations per cluster, repeated 40 times with 10 s intertrain interval). rTMS was conducted for 7 consecutive days. Starting from 3 days before the rTMS procedure, the mice were habituated to the coil for 10 min each day. For sham stimulation of the control group, the coil was placed immediately above the skull without magnetic stimulation. For electric field simulation, the open accessed electromagnetic simulation software (Simnibs) was used as described previously^31,32^. The coil inductance was approximately 17 µH, the capacitance was 140 µF, the reference voltage was 1,600 V and the reference frequency was 3,250 Hz. The maximum current change rate of the coil was approximately 9.4e7 A s^−1^. According to the TMS stimulating intensity, the current change rate was set at approximately 40% of the maximum current change rate.

#### In vitro rTMS treatment

Five days before the migration test, cells received high-frequency (10 Hz) rTMS every 24 h (five times in total). The circular coil (Yiruide Co. Ltd.) were placed under the culture dish and the distance from the center of the coil to the bottom of the dish was 0.5 cm. The parameters of high-frequency rTMS were the same as the above animal stimulation protocol. For the control group, the coil was maintained at the bottom of the Petri dish without stimulation.

### Statistical analysis

All behavior analyses and statistics were performed by an investigator who was blinded to the experimental design. No sample calculation was performed. For the in vitro study, at least three batches of cells or organoids were used for each experiment. For the in vivo study, at least three mice were included in each group for morphological and biochemical analysis, and 9–12 mice were included in each group for the behavior analysis. Each behavior test was conducted using distinct groups of animals. Data are presented as the mean ± s.e.m. Normal distribution was assessed by the Shapiro–Wilk test. Statistical comparisons were made using a Student’s *t*-test or a one-way or two-way analysis of variance (ANOVA) with Student–Newman–Keuls post hoc analysis. A *P* value less than 0.05 was considered as statistically significant.

## Results

### Multiple toxicities of Sev exposure on hNPCs and human neurons in vitro

We first examined the effects of Sev on the proliferation and survival of hNPCs derived from two hES cell lines (H8 and H9), using a widely adopted clinically relevant procedure^2,33,34^ (Supplementary Fig. 1a). Sev was delivered shortly after rosette selection when over 80% of cells were Nestin positive or Pax6 positive (Supplementary Fig. 1b, c). In H8 hES cell-derived NPCs, no significant difference of Ki67-positive and TUNEL-positive cells were found between control and Sev-treated cells (Supplementary Fig. 1d–g). Propidium Iodide (PI)-labeled cells were rarely detected in Sev-treated cells (Supplementary Fig. 1h). Similar results were also obtained in H9 hES cell-derived NPCs (Supplementary Fig. 1i–m). These data are in agreement with previous observations that Sev does not influence the proliferation and survival of hNPCs in vitro^35^. Since H8 and H9 cells respond similarly to Sev, we adopted H8 hES cell-derived NPCs for all the following experiments.

**Fig. 1:**
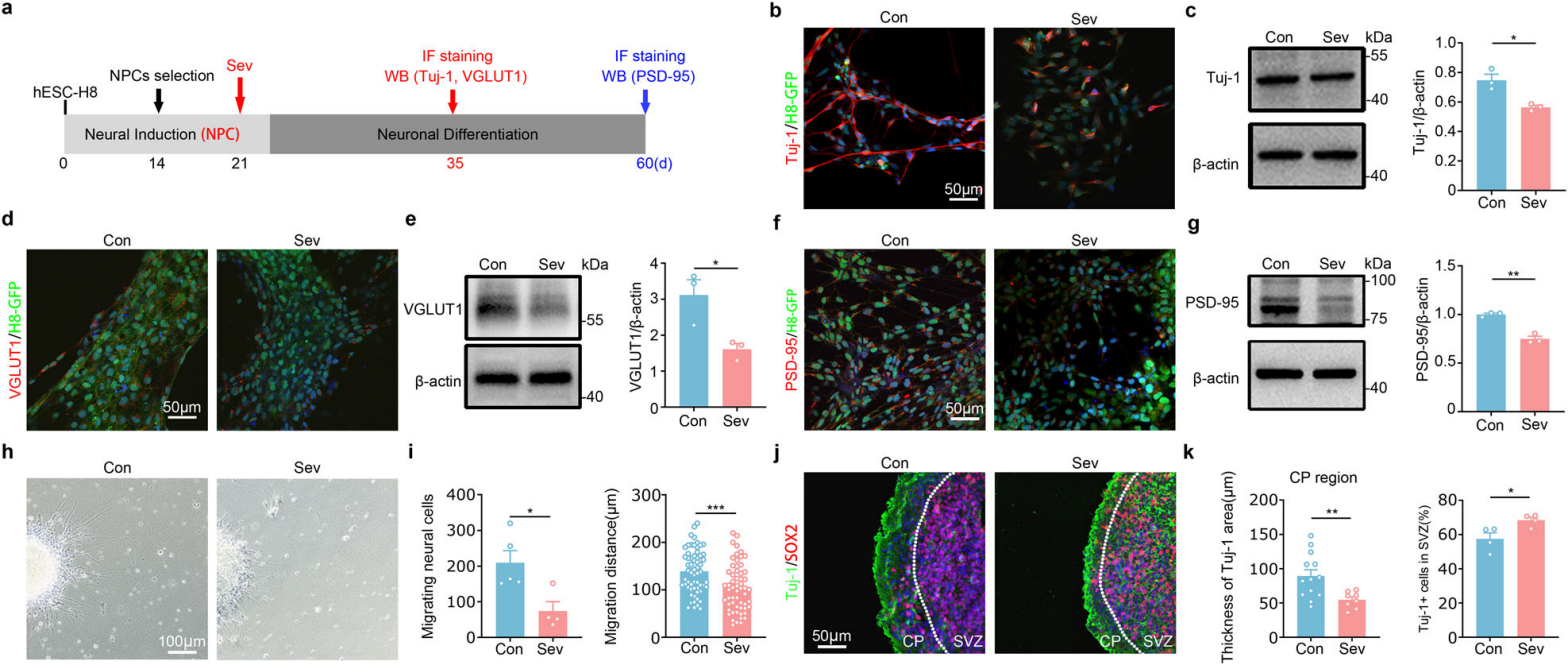
Toxic effects of Sev on hNPCs/neurons in vitro. **a** The experimental design for **b**–**g**. **b,** **c** Immunostaining (**b**) and western blotting (**c**) of Tuj-1 in hNPCs treated with or without Sev. **d,** **e** Immunostaining (**d**) and western blotting (**e**) of VGLUT1 in human neurons treated with or without Sev. **f,** **g** Immunostaining (**f**) and western blotting (**g**) of PSD-95 in human neurons treated with or without Sev. Notice the reduction of Tuj-1, VGLUT1 and PSD-95 following Sev treatment. **h**, **i** Representative images of migrating hNPCs from neurospheres treated with or without Sev (**h**) and the quantification of migrating cells and migrating distance (**i**). **j,** **k** Double immunostaining of Tuj-1/Sox2 in hCOs and the quantification of Tuj-1^+^ cells in the CP. Notice the suppression of human neuronal migration in Sev-treated neural spheres and hCOs. *N* = 3 batches of cells per group in **b**–**g** and 4–5 batches of cells per group in **h**–**k**. Student’s *t*-test. Error bars show s.e.m. *P < 0.05, **P < 0.01 and ***P < 0.001. Con control, IF immunofluorescence, WB western blot.

As Sev is reportedly toxic to neuronal differentiation and synaptic formation in rodents^33,36^, we examined whether this were the case in hNPCs (Fig. 1a). Immunocytochemistry and western blotting showed a significant reduction of Tuj-1, VGLUT1 and postsynaptic density protein 95 (PSD-95) following Sev treatment (Fig. 1b–g).

During development, most of the immature neurons migrate to their destined brain regions before forming the correct neural circuits. To explore whether Sev exposure influenced neuronal migration, Sev-pretreated hNPCs were suspended in culture until neurosphere formation and then seeded onto poly-d-lysine precoated dishes to let the neurons migrate. Notably, less neurons migrated out from Sev-pretreated neurospheres, as compared with the control group (Fig. 1h, i, left). The average migrating distance in Sev-pretreated cells was significantly shorter (Fig. 1i, right).

In Sev-treated hCOs, significantly less Tuj-1-positive and CTIP2-positive cells were seen in the cortical plate (CP), while more Tuj-1-positive and CTIP2-positive cells were found in subventricular zone (SVZ), as compared with control hCOs (Fig. 1j, k and Supplementary Fig. 1n). The number of Sox2-positive cells were similar between control and Sev-pretreated hCOs (Supplementary Fig. 1o). Together, these data indicate that Sev exposure inhibits human neuronal differentiation, synaptogenesis and migration in vitro.

### In vivo inhibition of human neuronal migration, but not differentiation and synaptogenesis, by Sev

**Fig. 2:**
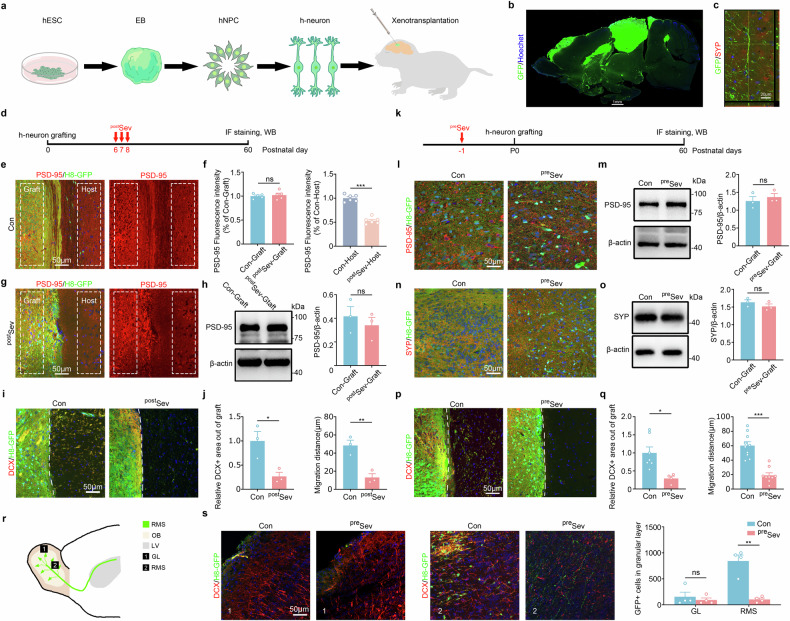
Inhibition of human neuronal migration by Sev in chimeric mice. **a** The experimental scheme for making human neuronal chimeric mice. **b** A representative sagittal image of human neuronal chimeric mice. **c** A representative image of double immunostaining of GFP with synaptophysin in human neuron chimeric mice. Notice the GFP-positive human neurons and long axons in mouse brain. **d** The experimental design for **e**–**j**. Sev was administered at P6–8. **e**–**h** Immunostaining and western blotting of PSD-95 in control and Sev-treated chimeric mice. Notice the reduction of PSD-95 immunoreactivity in Sev-treated host tissue, but not in Sev-treated hNPC graft. **i**, **j** Double immunostaining of DCX with GFP in control and Sev-treated chimeric mice (**i**) and quantification of out-migrated DCX^+^ cells (**j**). **k** The experimental design for **l**–**q**. hNPCs were pretreated by Sev before transplantation. **l**, **m** Immunostaining (**l**) and western blotting (**m**) of Tuj-1 in control and Sev-pretreated chimeric mice. **n,** **o** Immunostaining (**n**) and western blotting (**o**) of synaptophysin in control and Sev-pretreated chimeric mice. **p,** **q** Double immunostaining of DCX with GFP in control and Sev-pretreated chimeric mice and quantification of out-migrated DCX^+^ cells. Notice the reduction of out-migrated DCX^+^ cells in chimeric mice treated with Sev at postnatal stages (^post^Sev) or pretreated with Sev before hNPC transplantation (^pre^Sev). **r,** **s** A diagram of the image sampling region (**r**) and representative images of DCX/GFP-positive cells in the olfactory bulb of chimeric mice made from Sev-pretreated or normal hNPCs and the quantification (**s**). Notice the dramatic reduction of GFP-positive cells in the olfactory bulb of Sev-pretreated chimeric mice. *n* = 3–4 chimeric mice per group. Student’s *t*-test in **f,** **h,** **j,** **m,** **o** and **q** and one-way ANOVA in **s**. The error bars show s.e.m. *P < 0.05, **P < 0.01 and ***P < 0.001. Con control, IF immunofluorescence, LV lateral ventricle, OB olfactory bulb, WB western blot, RMS rostral migratory stream, GL glomerular layer.

To explore the effects of Sev on human neurons in vivo, we adopted a human–mouse chimeric model in which human neurons integrate well and keep species-specific properties in the mouse brain^23^. Human neuronal chimeric mice were made by transplanting H8 hES cell–NPCs into the forebrain cortex of neonatal NOD-SCID mice as described previously^37^ (Fig. 2a). GFP-positive human neurons survive well, grow very long axons and form human–human or human–mouse synapses in most forebrain regions (Fig. 2b, c).

We first adopted a widely used experimental paradigm in animal studies^38^, in which Sev was given to neonatal chimeric mice from postnatal day 6 to 8 (P6–8), and the neuronal phenotypes were evaluated in adults (Fig. 2d). Immunohistochemistry and western blotting showed similar levels of Tuj-1 and VGLUT1 in the hNPC grafts between Sev-treated chimeric mice and control chimeric mice (Supplementary Fig. 2a–e). In comparison with control chimeric mice, a significant reduction in PSD-95 immunoreactivity was detected in host tissues of Sev-treated chimeric mice, while no change in PSD-95 immunoreactivity was found in hNPC grafts of Sev-treated chimeric mice (Fig. 2e–g). Western blotting confirmed the similar levels of PSD-95 in hNPC grafts of Sev-treated mice and control chimeric mice (Fig. 2h).

In terms of cell migration, numerous DCX-positive cells were found migrating out of hNPC graft in control chimeric mice, while much fewer DCX-positive cells migrated out in Sev-treated chimeric mice (Fig. 2i, j). These data reveal the distinct phenotypes of hNPCs in vivo relative to in vitro.

To clarify whether these distinct results between in vivo and in vitro experiments were due to the different availability of Sev to cells in vivo versus in vitro, we made chimeric mice by Sev-pretreated hNPCs (Fig. 2k). No significant changes of human neuronal differentiation were found between Sev-pretreated and control chimeric mice, as evidenced by similar expression of Tuj-1, Tbr1, NKX2.1, CTIP2, DARPP32, GAD-67 and VGLUT1 in hNPC grafts (Supplementary Fig. 3a–j). In terms of synaptogenesis, immunostaining and western blotting of PSD-95 and synaptophysin in hNPC grafts, as well as synaptic densities along GFP-positive human axons, showed no significant difference between control and Sev-pretreated chimeric mice (Fig. 2l–o and Supplementary Fig. 4a). In addition, the spontaneous excitatory postsynaptic currents (EPSCs) of GFP-positive neurons in Sev-pretreated chimeric mice were similar to those in control chimeric mice (Supplementary Fig. 4b). These data indicate that the in vitro ‘toxic effects’ of Sev on human neuronal differentiation and synaptogenesis were not prominent in vivo. In contrast, significant less out migration of GFP/DCX-positive cells was found around the NPC graft, as well as the olfactory bulb in Sev-pretreated chimeric mice (Fig. 2p–s). Together, both the in vitro and in vivo data demonstrate a long-term impairment of human neuronal migration by Sev.

### Sev-induced deficiency of human neuronal migration impairs social function of chimeric mice

We next assessed whether Sev induced long-term behavior changes in chimeric mice. In the widely used postnatal exposure procedure, Sev-treated wild-type (WT) mice exhibited significant impairment in fear memory, a loss of interest in novel objects and enhanced anxiety in comparison with control WT mice (Supplementary Fig. 5), as previously reported^24^. Unexpectedly, Sev-treated chimeric mice and control chimeric mice showed similar behavior to naive WT mice in these tests (Supplementary Fig. 5). These data are in line with the above observation that Sev had no significant effects on human neuronal differentiation and synaptogenesis in vivo. Alternatively, integration of human neurons may counteract some of the harmful effects of Sev on mouse behavior.

**Fig. 3:**
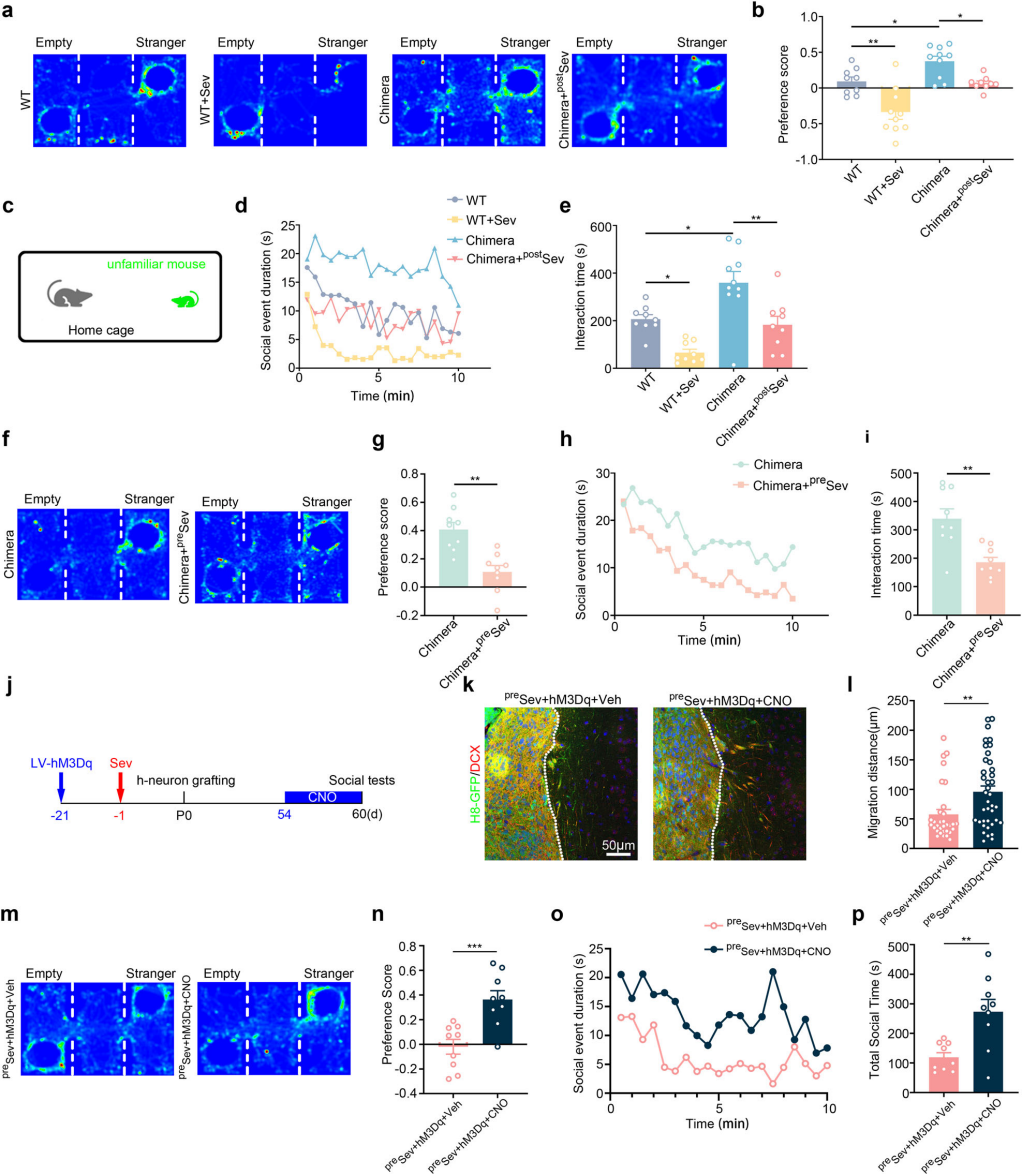
Social dysfunction of human neuronal chimeric mice exposed to Sev in early developmental stage. **a,** **b** A three-chamber test on WT mice, WT mice treated with Sev at postnatal stage (^post^Sev), control chimeric mice (chimera) and chimeric mice treated with Sev at the postnatal stage. **c**–**e** A resident–intruder assay on WT mice, WT mice treated with Sev at postnatal stage, control chimeric mice and chimeric mice treated with Sev at the postnatal stage. Notice the lower social preference and social interaction in chimeric mice treated with Sev at the postnatal stage. **f,** **g** A three-chamber test on control chimeric mice and chimeric mice made by Sev-pretreated hNPCs (^pre^Sev). **h,** **i** A resident–intruder assay on control chimeric mice and chimeric mice made by Sev-pretreated hNPCs. Notice the reduction of social preference scores and social interaction in chimeric mice made by Sev-pretreated hNPCs. **j** The experimental design for **k**–**p**. **k,** **l** Double immunostaining (**k**) and quantification (**l**) of GFP/DCX in chimeric mice derived from Sev-pretreated and hM3Dq-expressing hNPCs, which were treated with vehicle or CNO. CNO treatment significantly increased the migration of DCX/GFP-positive cells. **m,** **n** A three-chamber assay on chimeric mice derived from Sev-pretreated and hM3Dq-expressing hNPCs, which were treated with vehicle or CNO. **o,** **p** A resident–intruder assay on chimeric mice derived from Sev-pretreated and hM3Dq-expressing hNPCs, treated with vehicle or CNO. Notice the enhancement of social activity by CNO treatment. *N* = 6–9 mice per group. One-way ANOVA in **b** and **e** Student’s *t*-test in **g,** **i,** **l,** **n,** and **p**. *P < 0.05 and **P < 0.01. The error bars show s.e.m. Veh vehicle.

Interestingly, Sev-treated chimeric mice exhibited a dramatic reduction in social preference and social interaction compared with control chimeric mice (Fig. 3a–e). Given that Sev-treated WT mice also displayed less social preference, we next evaluated the social behavior of chimeric mice, which were made by Sev-pretreated hNPCs to exclude the effects of Sev on mouse neurons. The three-chamber assay and resident–intruder assay revealed an obvious reduction in social preference and social interaction in Sev-pretreated chimeric mice compared with control chimeric mice (Fig. 3f–i). Therefore, social dysfunction is the most predominant behavioral phenotype associated with Sev treatment in human neuronal chimeric mice.

To explore the relationship between human neuronal activity with social dysfunction in Sev-pretreated chimeric mice, we first assessed the response of human neurons to social stimulation by immunostaining of c-Fos. The results showed significantly less c-Fos-positive human neurons in Sev-pretreated chimeric mice compared with control chimeric mice (Supplementary Fig. 6a), indicating a low level of human neuronal activity in Sev-pretreated chimeric mice. To specifically activate human neurons, we adopted the hM3D(Gq)-chemogenetic system, in which CNO treatment could increase the neuronal activity of hM3D(Gq)-expressing cells. We made chimeric mice by using Sev-pretreated, hM3D(Gq)-expressing hNPCs (designated as ^Sev-hM3DGq^hNPC mice), and treated the ^Sev-hM3DGq^hNPC mice with CNO to activate human neurons and then performed morphological and social analyses (Fig. 3j). Immunohistochemistry showed that significantly more DCX-positive cells migrated out of the hNPC graft in CNO treated ^Sev-hM3DGq^hNPC mice compared with vehicle-treated ^Sev-hM3DGq^hNPC mice (Fig. 3k, l). The three-chamber and resident–intruder assays showed that CNO treatment significantly increased the social preference and social interaction activity of ^Sev-hM3DGq^hNPC mice (Fig. 3m–p). These data indicate that the deficiency of human neuronal migration may be associated with reduced neuronal activity and underlie the social dysfunction of Sev-pretreated chimeric mice.

### Sev exposure inhibits DVL-1/Ca^2+^ noncanonical Wnt signaling

**Fig. 4:**
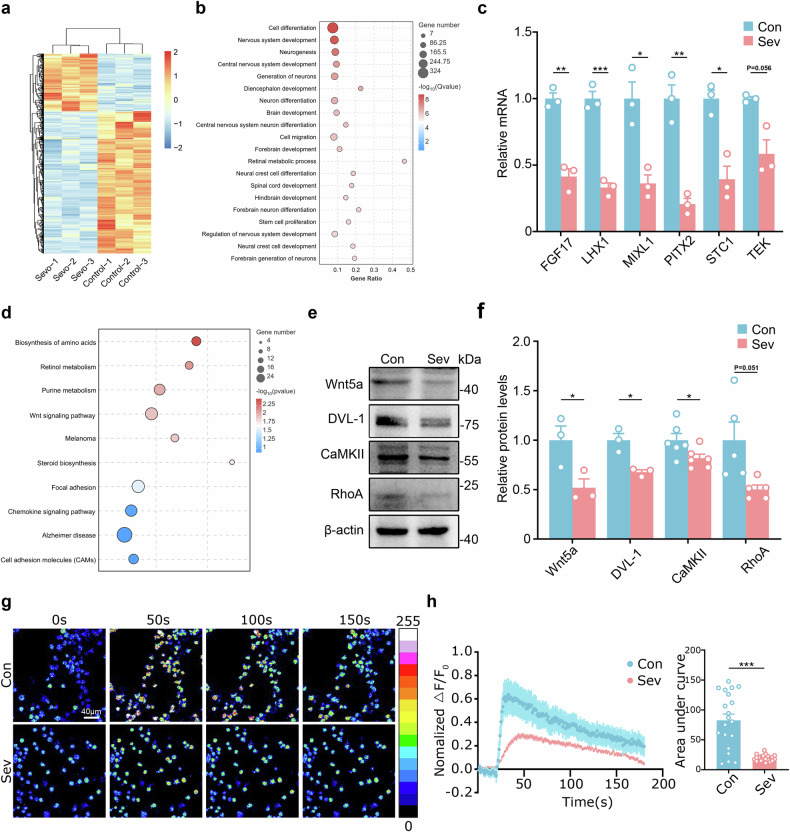
Downregulation of DVL-1/Ca^2+^ noncanonical Wnt signaling by Sev treatment. **a** A heat map of gene expression profiles of control and Sev-treated human neurons. **b,** **c** GO enrichment of the top 20 significantly changed biological processes (**b**) and qPCR verification of genes related to migration (**c**). **d** The top ten significantly changed signaling pathways. **e,** **f** Western blot validation of noncanonical Wnt signaling components in control and Sev-treated human neurons. **g,** **h** The calcium response of control and Sev-treated human neurons to KCl. Notice the significant downregulation of noncanonical Wnt signaling molecules and reduced calcium response in Sev-treated human neurons. *N* = 3 batches of cells. *P < 0.05 and **P < 0.01. One-way ANOVA in **c** and **f** and Student’s *t*-test in **h** (right). The error bars show s.e.m. Con control, DVL-1 disheveled-1, FGF17 fibroblast growth factor 17, LHX1 LIM homeobox protein 1, MIXL1 mix paired-like homeobox, PITX2 paired-like homeodomain 2, STC1 stanniocalcin 1, TEK TEK receptor tyrosine kinase.

To explore the underlying mechanism for the suppression of human neuronal migration by Sev treatment, we first tested whether blocking the GABA-A receptor could rescue human neuronal migration in vitro. The data showed that 3 days of treatment with bicuculline had no significant effect on hNPC migration (Supplementary Fig. 6b, c). We next compared the gene expression of control and Sev-treated hNPCs by RNA sequencing. A total of 308 genes were upregulated and 754 genes were downregulated in Sev-treated hNPCs (Fig. 4a). Gene Ontology (GO) analysis revealed that cell differentiation, neurogenesis and cell migration were among the top 20 significantly biological processes altered by Sev treatment (Fig. 4b). Among the top ten differential genes ranked by *P* values, three genes (*SERPINE2*, *STC1* and *CER1*) were related to cell migration, supporting cell migration as the prominent phenotype. Among the top ten significantly altered cell migration genes, six genes (*LHX1*, *Pitx2*, *MIXL1*, *FGF17*, *STC1* and *TEK*) were downregulated in Sev-treated hNPCs (Fig. 4c). These data further confirm the suppression of neuronal migration by Sev treatment.

The top ten significantly altered pathways, as determined by Kyoto Encyclopedia of Genes and Genomes analysis, included Wnt signaling (Fig. 4d), which participates in both neuronal differentiation and migration. Western blotting showed no significant change in canonical Wnt signaling components, such as β-catenin, pGSK3β (S9) and Axin2 (Supplementary Fig. 6d). In contrast, key noncanonical Wnt signaling molecules, such as Wnt5a, DVL-1, CaMKII and Rho-A, were all significantly lower in Sev-treated cells (Fig. 4e, f). Calcium imaging showed that Sev treatment remarkably inhibited Ca^2+^ responsiveness to KCl treatment (Fig. 4g, h).

Transcription profiling was carried out in vitro, and we further determined whether DVL-1 signaling was altered in Sev-pretreated chimeric mice. Significantly lower levels of DVL-1, CaMKII, Rho-A and Rac-1 were found in the hNPC grafts of Sev-pretreated chimeric mice than in control chimeric mice (Supplementary Fig. 6e). These data illustrate that Sev treatment inhibits DVL-1/Ca^2+^ noncanonical Wnt signaling in hNPCs.

### Activating DVL-1 signaling rescues both human neuronal migration and social function following Sev exposure

We next investigated whether activating noncanonical Wnt signaling could rescue the migration of Sev-treated hNPCs. In the cell migration assay, Wnt5a supplementation remarkably increased cell migration of Sev-treated hNPCs (Supplementary Fig. 7a, b).

**Fig. 5:**
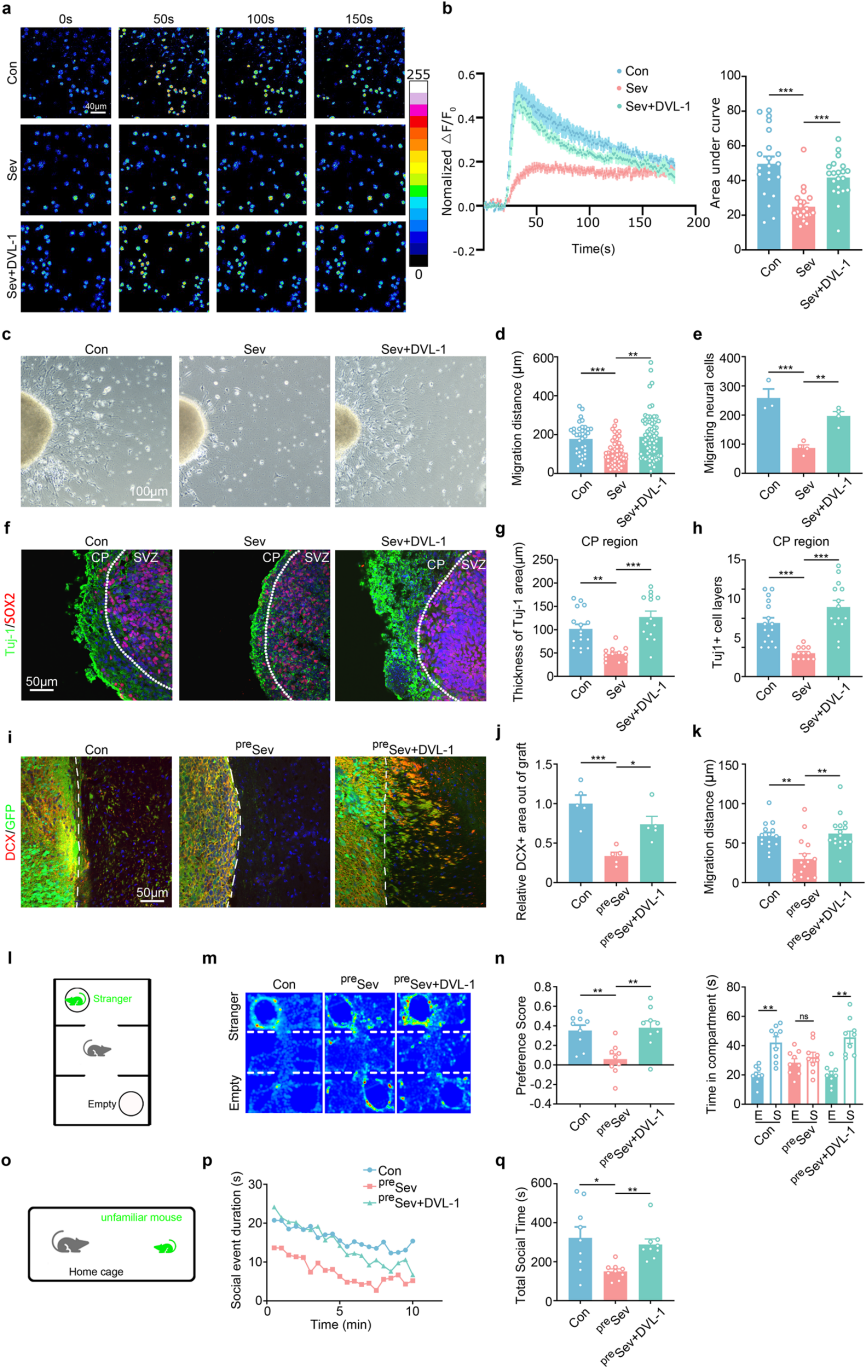
Effects of DVL-1 overexpression on the migration of Sev-pretreated hNPCs and social functions of Sev-pretreated chimeric mice. **a,** **b** The calcium response of control human neurons, Sev-pretreated human neurons and Sev-pretreated DVL-overexpressing (DVL-1^high^) human neurons to KCl stimulation. **c**–**e** Phase-contrast imaging (**c**) and quantification of cell migration in control human neurons, Sev-pretreated human neurons and Sev-pretreated DVL-1^high^ human neurons. **f**–**h** Double immunostaining of Tuj-1/Sox2 in control hCO, Sev-pretreated hCO and Sev-pretreated DVL-1^high^ hCO. Notice the restoration of human neuronal migration in the DVL-1^high^ culture. **i**–**k** Double immunostaining of GFP/DCX in control chimeric mice, Sev-pretreated chimeric mice (^pre^Sev) and Sev-pretreated DVL-1^high^ chimeric mice. Notice the recovery of migrating DCX-positive cells in Sev-treated DVL-1^high^ chimeric mice. **l**–**n** A three-chamber assay on control chimeric mice, Sev-pretreated chimeric mice and Sev-pretreated DVL-1^high^ chimeric mice. **o**–**q** A resident–intruder assay on control chimeric mice, Sev-pretreated chimeric mice and Sev-pretreated DVL-1^high^ chimeric mice. Notice the improved social activity in Sev-pretreated DVL-1^high^ chimeric mice. *N* = 3 batches of cells per group in **a**–**h** and 9–12 mice per group in **l**–**q**. *P < 0.05, **P < 0.01 and***P < 0.001. One-way ANOVA. Con, control; DVL-1, Disheveled-1.

Considering that DVL-1 connects multiple intracellular components of noncanonical Wnt signaling^39^, we infected hNPCs with a lentivirus expressing DVL-1 and obtained DVL-1^high^ hNPCs (Supplementary Fig. 7c), and then evaluated the intracellular Ca^2+^ response and cell migration following Sev treatment. DVL-1 overexpression increased the Ca^2+^ response of hNPCs upon Sev treatment as well as the expression levels of migratory genes (*FGF17*, *LHX1,* *PITX2* and *TEK*) (Fig. 5a, b and Supplementary Fig. 7d). In the cell migration assay, DVL-1^high^ hNPCs exhibited almost full recovery of hNPC migration after Sev exposure (Fig. 5c–e). In addition, DVL-1 overexpression significantly increased the out migration of Tuj-1-positive cells in Sev-pretreated human cerebral organoids (Fig. 5f–h).

To assess whether DVL-1 overexpression could alleviate the Sev-impaired neuronal migration and social function in chimeric mice, we treated DVL-1^high^ or control hNPCs with Sev, and then made chimeric mice using these hNPCs. A significantly larger number of GFP/DCX-positive cells migrated out in a longer distance in Sev-pretreated DVL-1^high^ hNPC chimeric mice than in Sev-pretreated control chimeric mice (Fig. 5i–k). The three-chamber assay showed that chimeric mice made from Sev-pretreated DVL-1^high^ hNPCs spent much more time in the social chamber compared with Sev-pretreated chimeric mice (Fig. 5l–n). The resident–intruder assay showed that chimeric mice made from Sev-pretreated DVL-1^high^ hNPCs interacted actively with intruder mice, at a similar level to the normal control mice (Fig. 5o–q). These data demonstrate that upregulating DVL-1 could alleviate the toxic effects of Sev on human neuronal migration and the corresponding social dysfunction.

### rTMS activates DVL-1 signaling and restores human neuronal migration post-Sev exposure

**Fig. 6:**
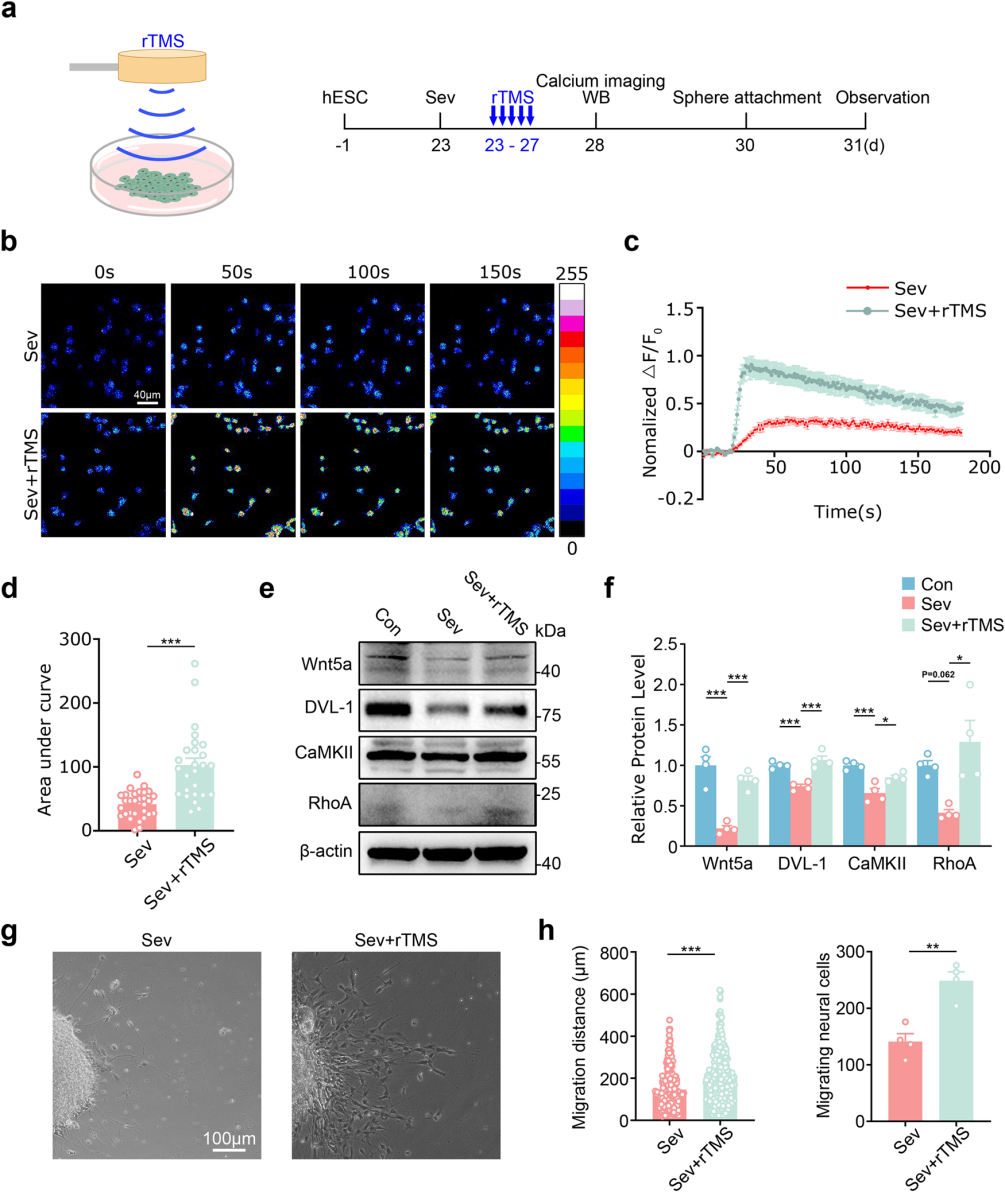
Effects of rTMS on cell migration and DVL-1/Ca^2+^ signaling of Sev-pretreated hNPCs in vitro. **a** The experimental design for **b**–**h**. **b**–**d** Ca^2+^ imaging of hNPCs treated with Sev or Sev plus rTMS, and the quantification. **e,** **f** Western blotting of Wnt5a, DVL-1, CaMKII and Rho-A in hNPCs treated with Sev or Sev plus rTMS (**e**), and the quantification (**f**). **g,** **h** Phase-contrast imaging (**g**) and quantification (**h**) of cell migration in hNPCs treated with Sev or Sev plus rTMS. *N* = 3 batches of cells. Student’s *t*-test in **d** and **h** and one-way ANOVA in **f**. *P < 0.05, **P < 0.01 and ***P < 0.001. Con control, WB western blot.

Since chemogenetic activation of human neurons could rescue the Sev-impaired neuronal migration, we speculated that rTMS, which can modulate neuronal activity in a noninvasive way, might be beneficial. To test this hypothesis, we first stimulated Sev-pretreated hNPCs with 10 Hz rTMS, which activates neuronal activity, and then evaluated Ca^2+^ responsiveness and cell migration in vitro (Fig. 6a). The results showed that rTMS treatment robustly increased the Ca^2+^ responsiveness of Sev pretreated cells (Fig. 6b–d). In addition, the protein levels of Wnt5a, DVL-1, CaMKII and Rho-A were recovered in rTMS-treated cells (Fig. 6e, f). Furthermore, hNPC migration was significantly enhanced in rTMS-treated cells (Fig. 6g, h).

**Fig. 7:**
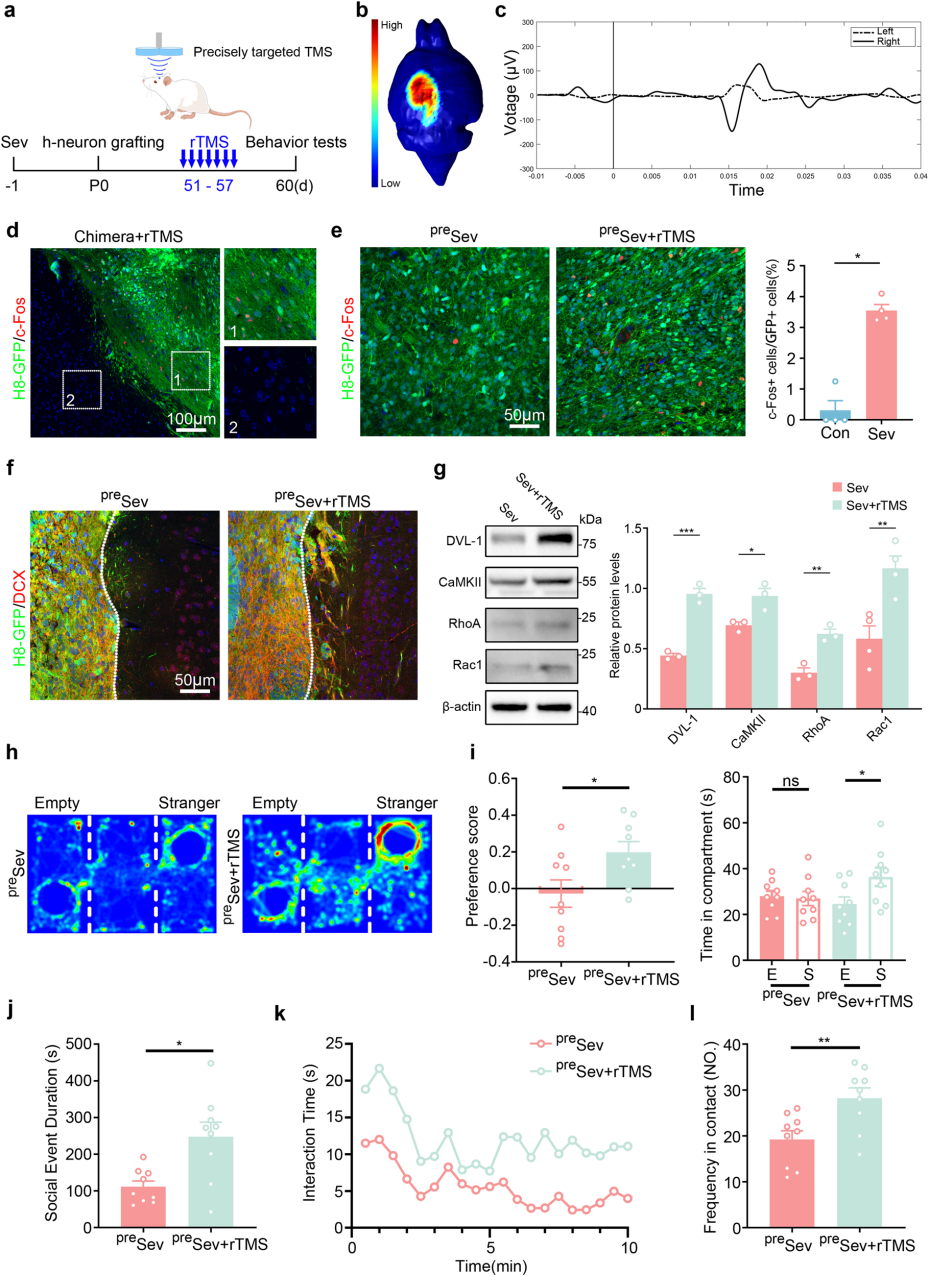
Improvement of human neuronal migration and social function in Sev-pretreated chimeric mice by precisely targeted rTMS. **a** The experimental scheme for **e**–**l**. **b**, **c** Stimulation of the left M1 region by precisely targeting rTMS (**b**) and the corresponding electromyographic activity (**c**). **d** Double immunostaining of c-Fos/GFP in an hNPC graft after rTMS treatment. Notice the c-Fos expression in the hNPC graft but not in mouse brain tissue. **e** Double immunostaining of c-Fos/GFP in an hNPC graft of Sev-pretreated chimeric mice (^pre^Sev) that had been treated with rTMS or not, and quantification. **f** Double immunostaining of DCX/GFP in Sev-pretreated chimeric mice with or without rTMS treatment. rTMS treatment increased the migration of DCX^+^ cells. **g** Western blotting of DVL-1, CaMKII, Rho-A and Rac-1 in Sev-pretreated chimeric mice with or without rTMS treatment. **h**–**j** A three-chamber assay on Sev-pretreated chimeric mice with or without rTMS treatment. **k**–**l** A resident–intruder assay on Sev-pretreated chimeric mice with or without rTMS treatment. Notice the improvement of social preference and social interaction by rTMS treatment. *N* = 3–4 mice per group in **c**–**g** and 8–10 mice per group in **f**–**l**. Student’s *t*-test in **i** (left), **j** and **l** and one-way ANOVA in **g** and **i** (right). *P < 0.05, **P < 0.01 and ***P < 0.001. Con control.

We next examined whether rTMS treatment could rescue the neuronal phenotype in vivo. To achieve this, we stimulated an hNPC graft of Sev-pretreated chimeric mice with a newly developed rTMS device, which could focus the magnetic field within a size of 0.5–1.5 mm^3^ (Fig. 7a, b). We first tested the region specificity of this precisely targeted TMS by placing the coil on the surface of the left motor cortex. Right hindlimb movement was triggered immediately following TMS stimulation without affecting the movement of left hindlimb (Supplementary Video 1). Electromyography recording showed that typical movement evoked potential in the right hindlimb (but not in left hindlimb) upon TMS stimulation (Fig. 7c), illustrating the brain region specificity of this precisely targeted rTMS.

Then, we stimulated the brain region of hNPC transplantation by the precisely targeted rTMS and validated the hNPC graft specificity by immunostaining of c-Fos immediately after rTMS treatment. c-Fos immunoreactivities were observed within the hNPC graft, but not in adjacent mouse brain tissue, confirming the specificity of TMS treatment (Fig. 7d). In comparison with sham treatment, the number of c-Fos-positive human neurons increased significantly upon rTMS treatment (Fig. 7e). In addition, many more DCX^+^ human neurons were found migrated out in rTMS-treated chimeric mice (Fig. 7f and Supplementary Fig. 7e). Western blotting revealed significant upregulation of DVL-1, Rho-A and Rac-1 in hNPC grafts by rTMS treatment (Fig. 7g). Further, the three-chamber and resident–intruder assays showed that rTMS treatment significantly improved the social preference and social interaction activity of Sev-pretreated chimeric mice (Fig. 7h–l). Together, these data demonstrate that rTMS modulation may be beneficial for rescuing the abnormal phenotypes of Sev-pretreated human neurons.

## Discussion

In the present study, by using two hES cell lines, hCOs and hNPC chimeric mice, we investigated the effects of Sev exposure on the survival, proliferation, differentiation, migration and synaptogenesis of hNPCs/human neurons, and the long-term behavior of Sev-pretreated human neuronal chimeric mice. Our data convincingly demonstrated an inhibition of human neuronal migration and subsequent social dysfunction of chimeric mice by Sev exposure. Further analysis revealed DVL-1-mediated noncanonical Wnt signaling as the key mechanism for this defect. Using a newly developed rTMS device that could specifically modulate human neural tissues, we showed that rTMS treatment was able to counteract the toxic effects of Sev on human neurons and subsequent behavioral outcomes in chimeric mice.

This study aimed to dissect the toxic effects of clinically relevant Sev exposure on human neurons. Recently, distinct results on the neural toxicity of Sev have been reported from two Mayo Anesthesia Safety in Kids studies, implying a gap between nonhuman primates and humans^40^. As our data were obtained directly from human neural cells, hCOs and humanized mice, the results are more relevant to what happens in clinic. Given that the results of in vitro experiments could not be compared with those of in vivo experiments, we put more emphasis on the in vivo data. Our observations imply that an in vivo microenvironment should be considered when evaluating the outcome of anesthetics exposure.

Surprisingly, a notable inhibition of neuronal migration and impairment of social function were consistently observed following Sev treatment, particularly in vivo. As neuronal migration is closely associated with their intrinsic electric activity^41^, the impairment of neuronal migration may be a delayed effect of neuronal inhibition by Sev. As far as we know, only one paper has mentioned inhibition of neuronal migration and one group reported social dysfunction following Sev exposure in mice^42,43^. Recently, anesthesiologists have noticed a correlation between early anesthetic exposure and long-lasting socio-affective behavior impairments^44,45^. Data from postmortem infant human brains indicated that neuronal migration toward forebrain regions is important for social behavior and executive function^46^, supporting our observation. In addition, abnormal neuronal migration is closely associated with autism spectrum disorder, a developmental psychological disease featuring social dysfunction^47,48^. Our data showed that stimulating neuronal migration, either by chemogenetic modulation of human neurons or by overexpressing DVL-1 in human neurons, effectively improved the social function of Sev-pretreated chimeric mice, substantiating the role of neuronal migration in Sev-induced social dysfunction and thus revealing an overlooked link between early general anesthesia and neuronal migration.

In general, Sev functions through a rapidly activating GABA receptor. The invalidation of a GABA-A receptor antagonist indicated that what we found was the long-term toxicity of early Sev exposure. Many previous studies, including ours^24^, have documented long-term inhibition of canonical Wnt signaling by Sev at the postnatal stage^49–51^. In the present study, we observed delayed downregulation of DVL-1/Ca^2+^ noncanonical Wnt signaling. It is known that noncanonical Wnt signaling plays important roles in synaptogenesis, axon guidance and neuronal migration^52,53^. As an intracellular hub of noncanonical Wnt signaling, DVL-1 activates CaMKII/Ca^2+^ signaling^54^ to direct the formation of lamellipodia and filopodia^55^, small GTPases (Rho-A and Rac) to modify cytoskeleton architecture and Cdc42 to modulate cell junctions^39^. The changes in CaMKII/Ca^2+^ and Rho-A/Rac-1 in our experimental setting strongly indicate that DVL-1-mediated noncanonical Wnt signaling serves as a therapeutic target for Sev-associated toxicity to human neurons.

More importantly, our data suggested a clinically applicable treatment for this Sev-induced long-term toxicity, namely rTMS. Owing to its noninvasiveness, rTMS has been successfully applied to mood-defective disorders^56^. Previous animal studies, including ours, have demonstrated that rTMS modulation could improve social function in autism spectrum disorders^57^. However, in most animal studies, the magnetic field covers the whole cortex, leaving the targeted area unspecific. Based on a two-coil TMS transducer that could rapidly and accurately adjust the orientation of the electric field^58^, we developed a three-coil TMS transducer that could confine the magnetic field within a size of 0.5–1.5 mm^3^. After validating the brain region specificity of this precisely targeting rTMS, we demonstrated that rTMS treatment not only restored neuronal migration, but also reversed DVL-1/Ca^2+^ signaling. These data indicated that TMS may lead to a chronic change of intracellular signaling besides instant modulating neuronal activity.

Together, our data indicate that neuronal migration and related long-term social dysfunction should be considered in the clinical applications of Sev to children and pregnant woman. Activating DVL-1-mediated noncanonical Wnt signaling or specific rTMS treatment may be beneficial for attenuating this toxicity.

## Supplementary information

Supplementary information.

Full gel images of main figures.

Full gel images of supplementary figures.

Statistic methods for each figures.

Supplementary Video 1.

## Data Availability

The datasets used and/or analyzed during the current study are available from the corresponding author on reasonable request.
