# Supplementary material for: Human neuron chimeric mice reveal impairment of DVL-1-mediated neuronal migration by sevoflurane and potential treatment by rTMS
Source: Exp Mol Med. 2025 Apr 1;57(4):745–58. doi: 10.1038/s12276-025-01425-0 (PMC12045952; doi:10.1038/s12276-025-01425-0)
Supplement: Supplementary file 1 — Supplementary information. [file 12276_2025_1425_MOESM1_ESM.pdf]

Title: Human neuron chimeric mice reveal impairment of DVL-1-mediated neuronal migration by Sevoflurane and potential treatment by rTMS

Running title: Authentic toxicity of Sevoflurane on human neurons

Authors: Youyi Zhao[1,2†], Ya Zhao[3†], Lirong Liang[1†], Andi Chen[2], Yuqian Li[2], Ke Liu[3], Rougang Xie[2], Honghui Mao[2], Boyang Ren[2], Bosong Huang[2], Changhong Shi[3], Zhicheng Shao[4], Shengxi Wu[2]*, Yazhou Wang[1]*, Hui Zhang[1]*

Supplementary information

Supplementary figure legends:

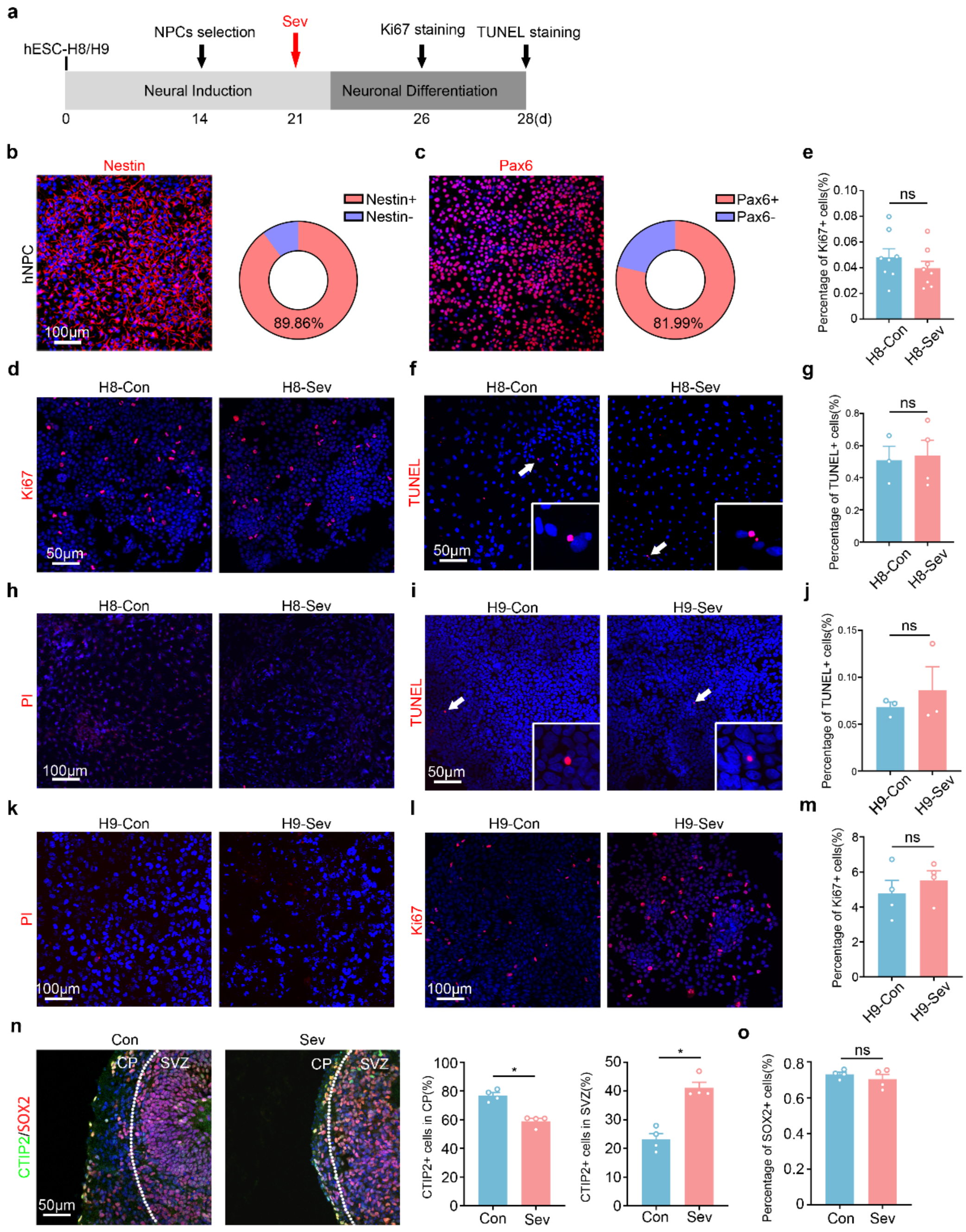

Supplementary Figure-1. Effects of Sev on the proliferation and survival of two human embryonic cell lines and neuronal migration in hCO.

(a) Experimental design for “b-m”. (b, c) Immunostaining and quantification of Nestin and Pax6 in H8-derived NPCs. (d-h) Immunostaining of Ki67, TUNEL staining and

PI-labeling in ESC-H8-derived hNPCs after Sev treatment and the corresponding quantification. (i-m) Immunostaining of Ki67, TUNEL staining and PI-labeling in ESC-H9-derived NPCs after Sev treatment and the corresponding quantification. Arrows point to TUNEL-positive cells. (n, o) Double-immunostaining of CTIP/Sox2 in hCO pretreated with or without Sev and the corresponding quantification. N = 3-4 batches of cells. Students' *t* test. *$P<0.05$. IF, immunoflurosence. hESC, human embryonic stem cell. hNPC, human neural progenitor cells. Con, control. Sev, Sevoflurane. CP, cortical plate, SVZ, subventricular zone. ns, no significance.

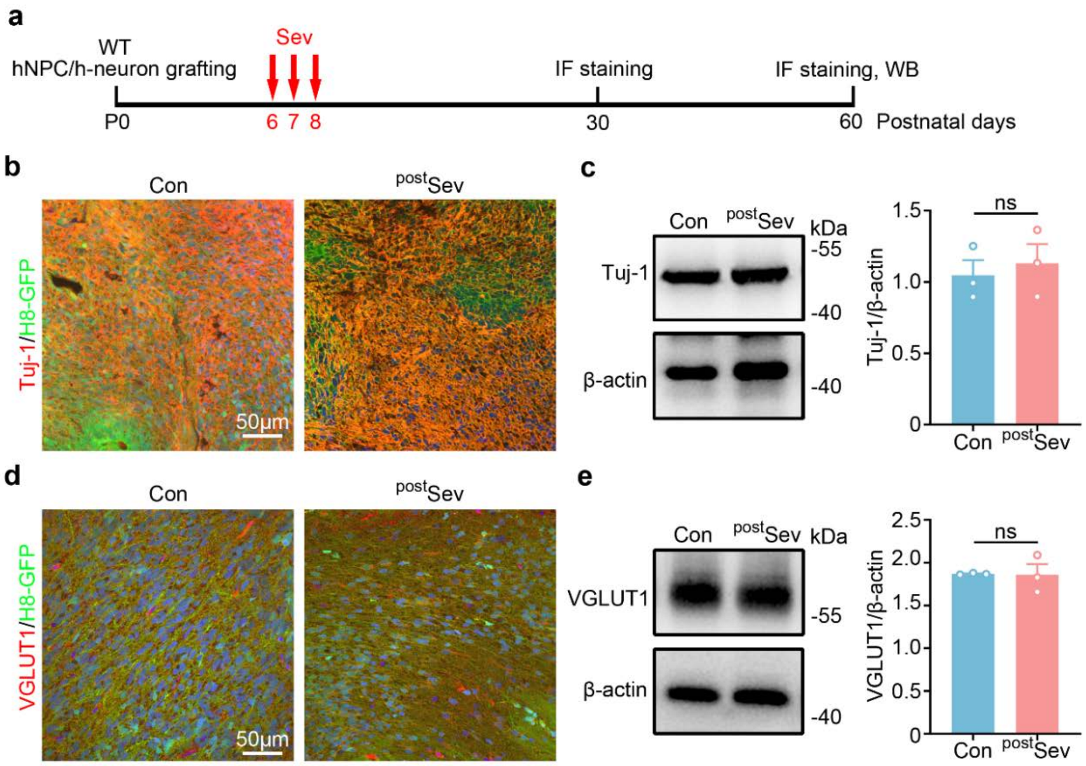

Supplementary Figure-2. Effects of postnatal Sev-treatment on the differentiation of hNPCs in chimeric mice.

(a) Experimental design. (**b, c)** Double-staining of Tuj1/GFP, and Western-blotting of Tuj1 in grafts of control and Sev-treated chimeric mice. (**d, e)** Double-staining of VGLUT1/GFP, and Western-blotting of VGLUT1 in grafts of control and Sev-treated chimeric mice. N = 3 mice per group. Students' *t* test. IF, immunoflurosence. Con, control. Sev, Sevoflurane. ^{Post}Sev, Sev treatment at postnatal stage. WB, western-blot. WT, wild type.

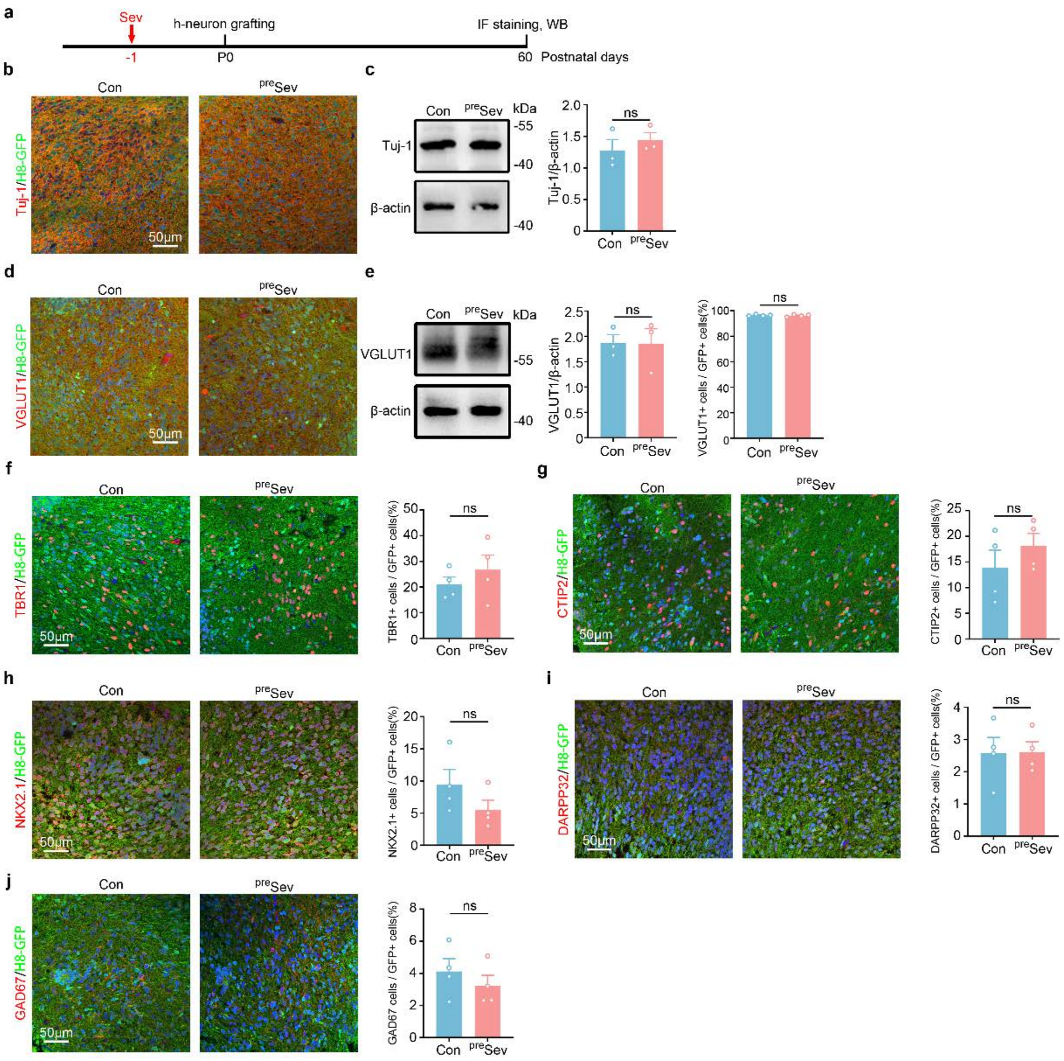

Supplementary Figure-3. Effects of Sev-pretreatment on the differentiation of hNPCs in chimeric mice.

(a) Experimental design. (**b, c)** Double-staining of Tuj1/GFP, and Western-blotting of Tuj1 in grafts of control and Sev-pretreated chimeric mice. (**d, e)** Double-staining of VGLUT1/GFP, and Western-blotting of VGLUT1 in grafts of control and Sev-pretreated chimeric mice. (f-j) Double-staining of Tbr1/GFP, NKX2.1/GFP, GAD67/GFP, CTIP2/GFP, DARPP32/GFP, in grafts of control and Sev-pretreated chimeric mice, and quantification. N = 3-4 mice per group. Students' *t* test. IF,

immunofluorescence. Con, control. Sev, Sevoflurane. ^{Pre}Sev, Sev pretreatment of hNPC. WB, western-blot. WT, wild type. ns, no significance.

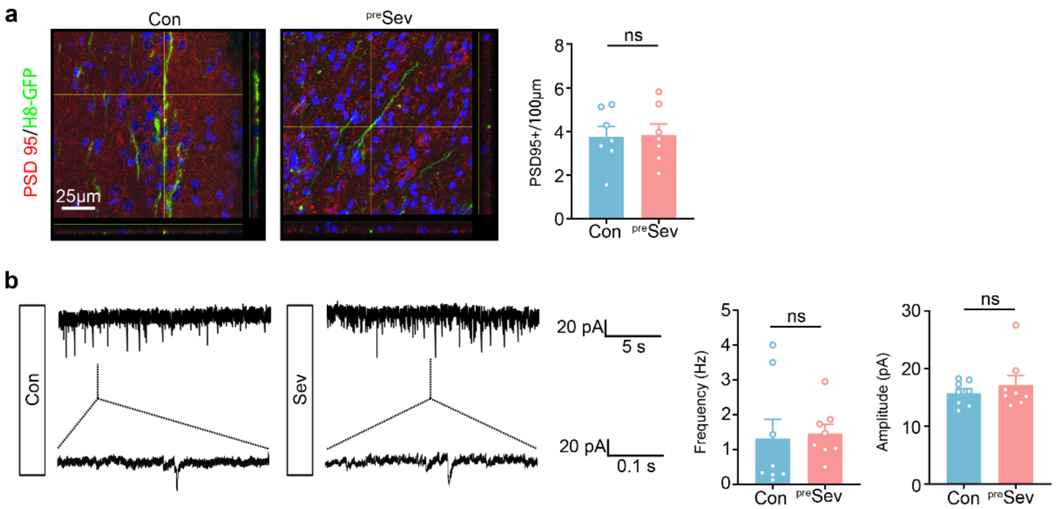

Supplementary Figure-4. Effects of Sev-pretreatment on synaptogenesis of human neurons in chimeric mice.

(a) Double-staining of PSD-95/GFP in grafts of control and Sev-pretreated chimeric mice, and quantification. (b) Patch-clamp recording to spontaneous synaptic activity in human neurons of control and Sev-pretreated chimeric mice, and quantification. N = 6 mice per group in (a), 6-8 neurons from 2 mice in (b). Students' *t* test. Con, control. Sev, Sevoflurane. ^{Pre}Sev, Sev pretreatment of hNPC. ns, no significance.

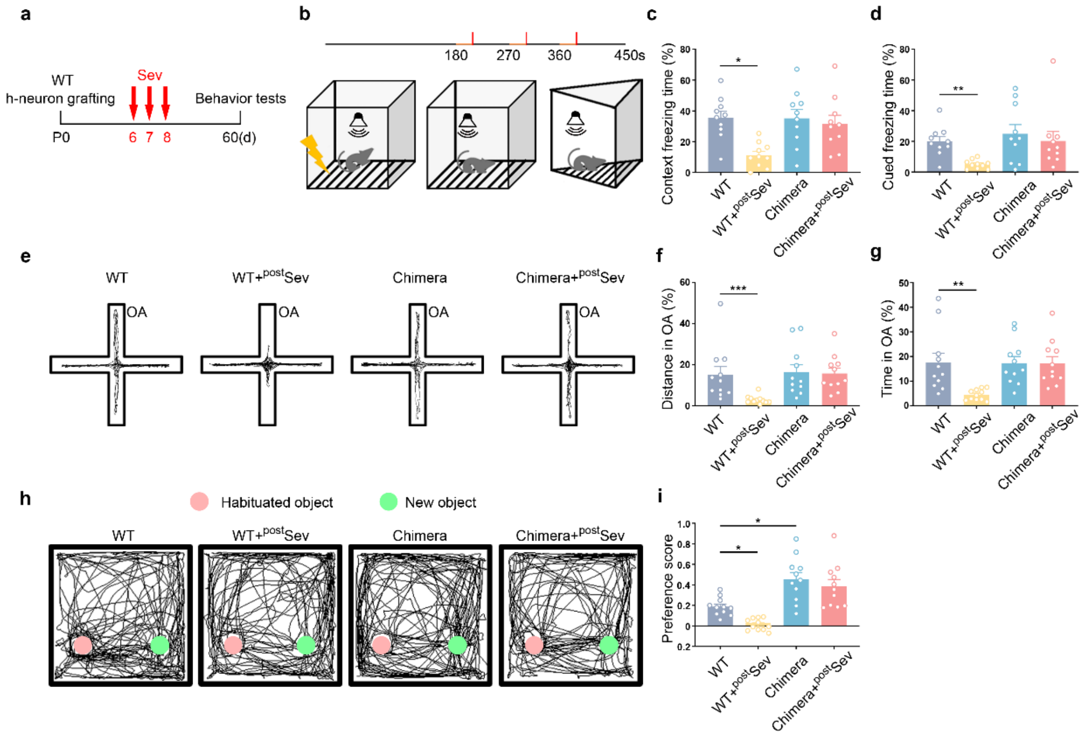

Supplementary Figure-5. Effects of postnatal Sev-treatment on the cognitive function of chimeric mice.

(a) Experimental design for “b-i”. (b-d) Fear conditioning test of wild type (WT) mice, WT mice treated with Sev at postnatal stage (^{post}Sev), control chimeric mice (chimera), and chimeric mice treated with Sev at postnatal stage. (e-g) Elevated Plus Maze assay of WT mice, WT mice treated with Sev at postnatal stage, control chimeric mice, and chimeric mice treated with Sev at postnatal stage. (h, i) Novel object exploration test of WT mice, WT mice treated with Sev at postnatal stage, control chimeric mice, and chimeric mice treated with Sev at postnatal stage. N = 9-11 mice per group. Two-way ANOVA. *P<0.05. **P<0.01.***P<0.001. Con, control. Sev, Sevoflurane. ^{Post}Sev, Sev treatment at postnatal stage.

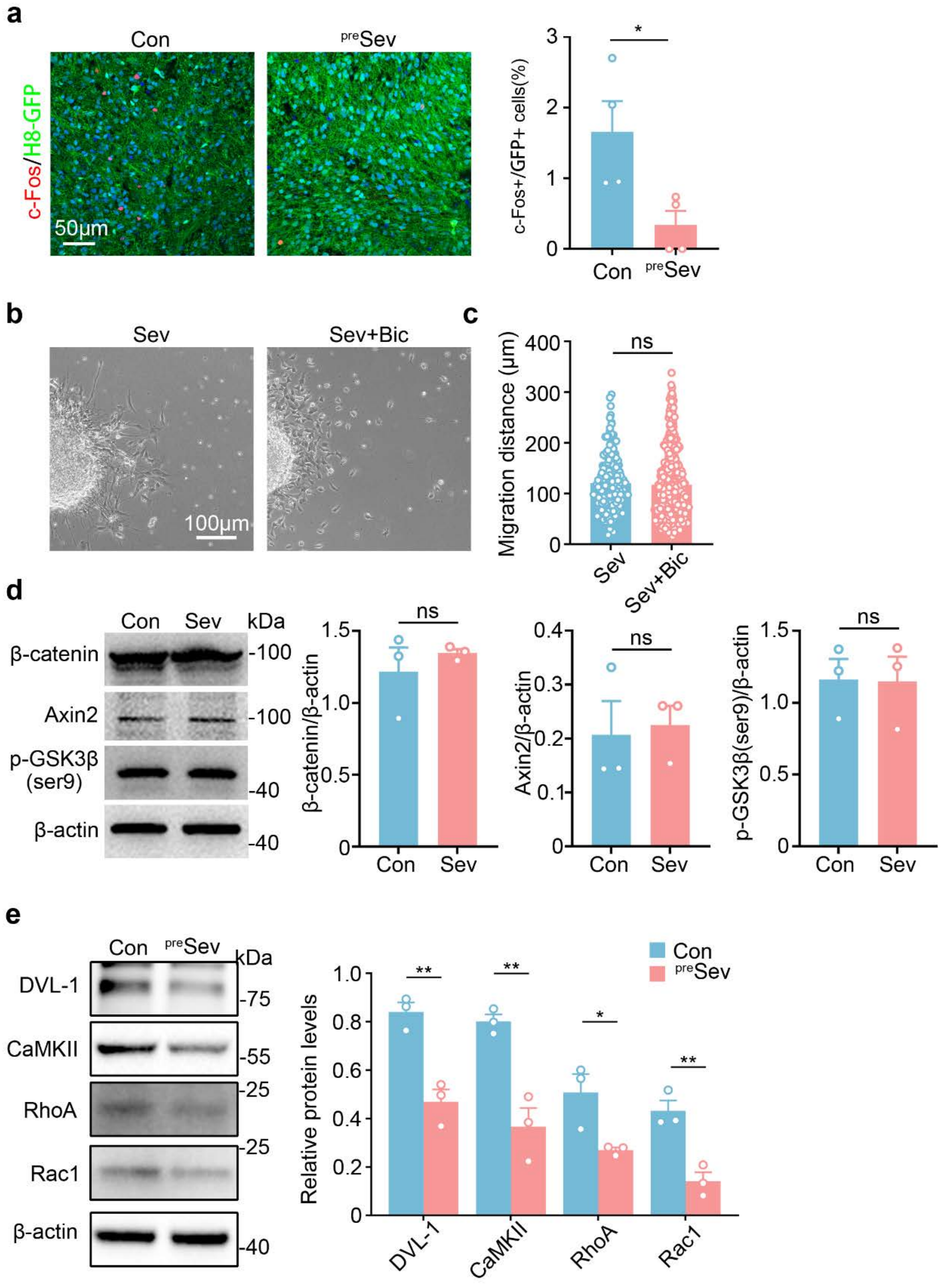

Supplementary Figure-6. Effects of Sev-pretreatment on human neuronal activity and Wnt signaling in hNPCs.

(a) Double-staining of c-Fos/GFP in grafts of control and Sev-pretreated chimeric mice,

and quantification. (b, c) Cell migration of hNPCs pretreated with Sev, or combination of Sev and Bicuculline. (d) Western-blotting of β-catenin, Axin2 and pGSK-3β(S9) in control and Sev treated hNPCs. (e) Western-blotting of DVL-1, CaMKII, Rho-A and Rac-1 in grafts of control and Sev-pretreated chimeric mice, and quantification. Students' *t* test in (a-d), one way ANOVA in (e). N = 3-4 mice per group in (a, e), 3 batches of cells in (b-d). $*P<0.05$. $**P<0.01$. Con, control. Sev, Sevoflurane. Bic, Bicuculline. ^{Pre}Sev, Sev pretreatment of hNPC. ns, no significance.

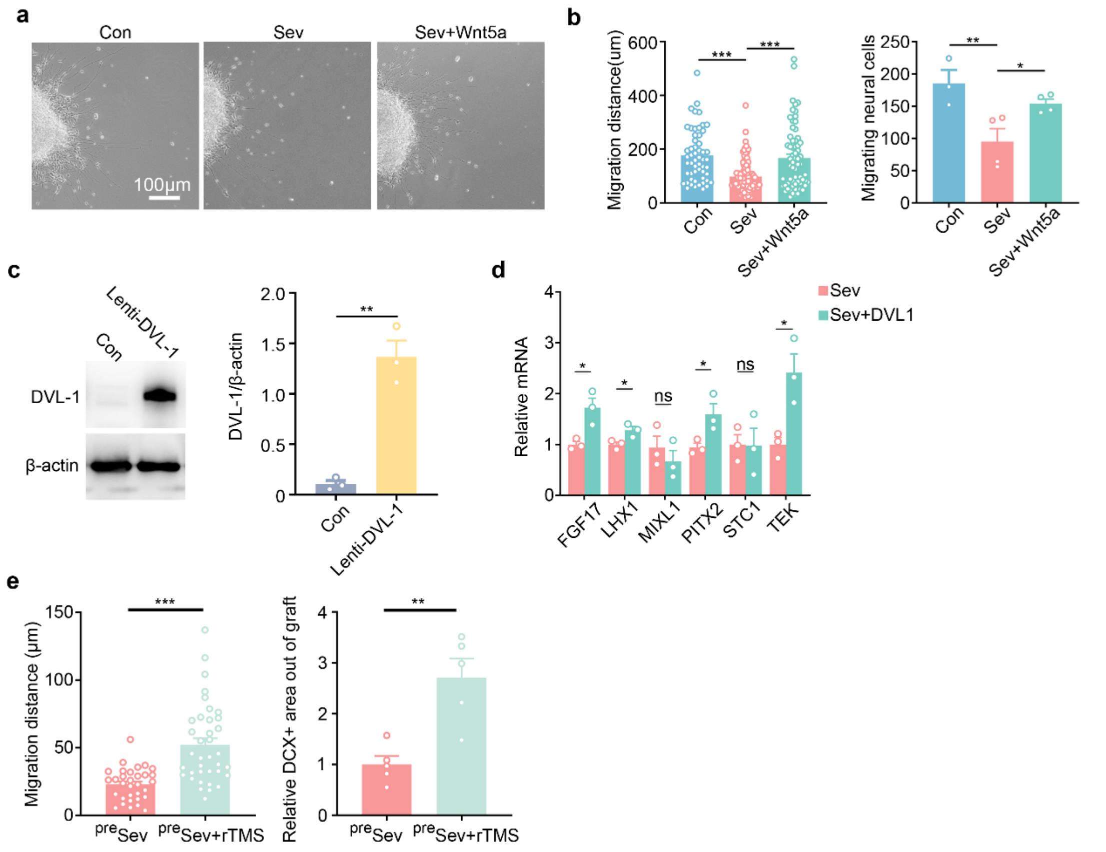

Supplementary Figure-7. Restoration of human neuronal migration by DVL-1 non-canonical Wnt signaling and rTMS

(a, b) Phase-contrast imaging and quantification of migrating human neurons in control neurospheres, Sev-pretreated neurospheres, and Sev-pretreated neurospheres with Wnt5a supplement. (c)Western-blotting of DVL-1 in control hNPCs and Lenti-DVL-1 infected NPCs. (d) qPCR of cell migrating genes (*FGF17*, *LHX1*, *MIXL1*, *PITX2*, *STC1*, *TEK*) in Sev-pretreated hNPCs with or without DVL-1 overexpression. (e) quantification of migrating cells in Fig-7f. One-way ANOVA in (b, d). Students' *t* test in (c, e). N = 3 batches of cells per group in (a-d), 5 mice per group in (e). *P<0.05. **P<0.01. ***P<0.001. Con, control. Sev, Sevoflurane.

[Pre]Sev, Sev pretreatment of hNPC. ns, no significance.

Supplementary video 1: Supplementary video corresponds to Fig. 7b-c showing the movement of right hindlimb upon TMS stimulation of left motor cortex.
