# Supplementary figures and images for: Human neuron chimeric mice reveal impairment of DVL-1-mediated neuronal migration by sevoflurane and potential treatment by rTMS
Source: Exp Mol Med. 2025 Apr 1;57(4):745–58. doi: 10.1038/s12276-025-01425-0 (PMC12045952; doi:10.1038/s12276-025-01425-0)

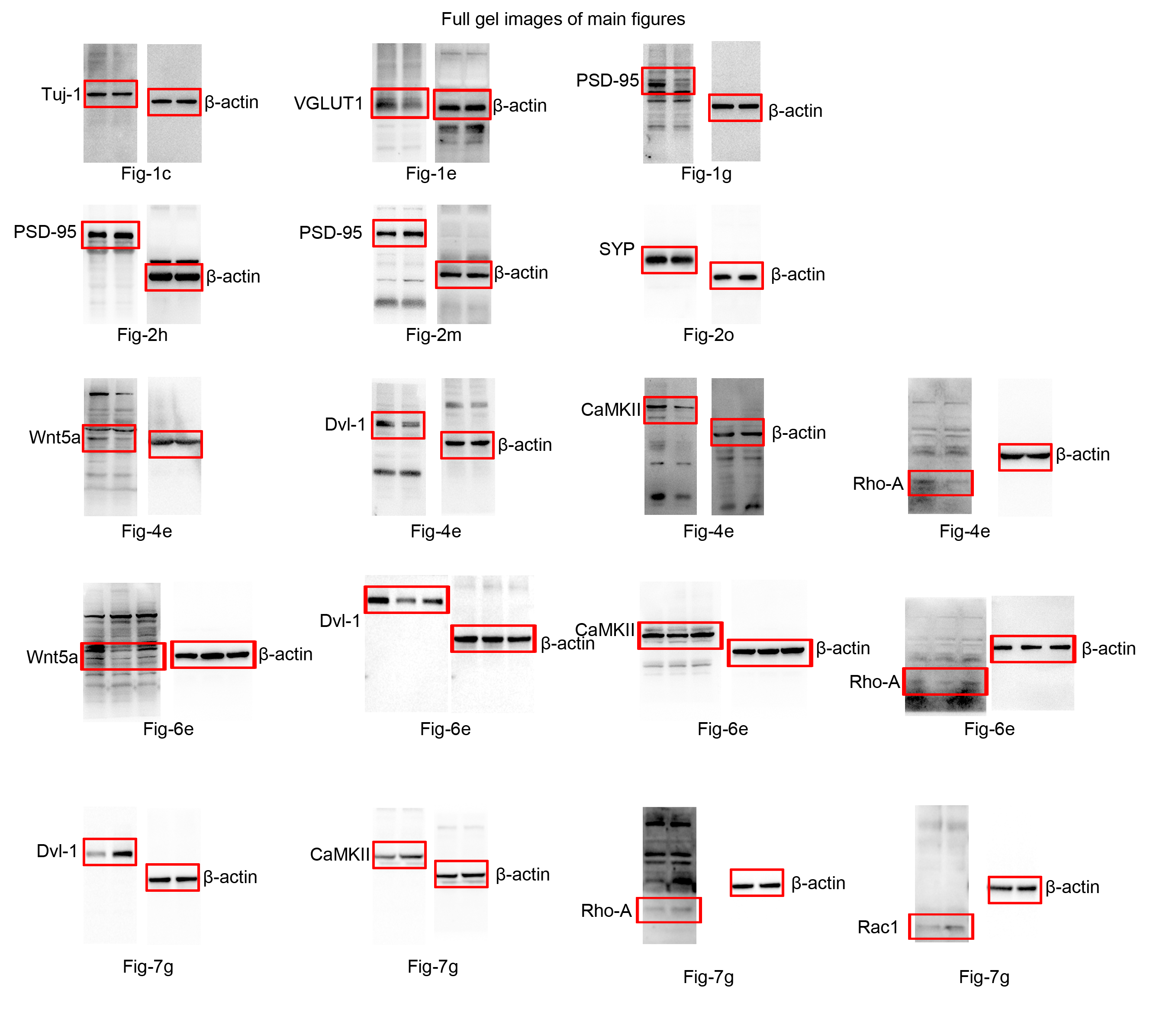

Supplement: Supplementary file 2 — Full gel images of main figures. [file 12276_2025_1425_MOESM2_ESM.tif]

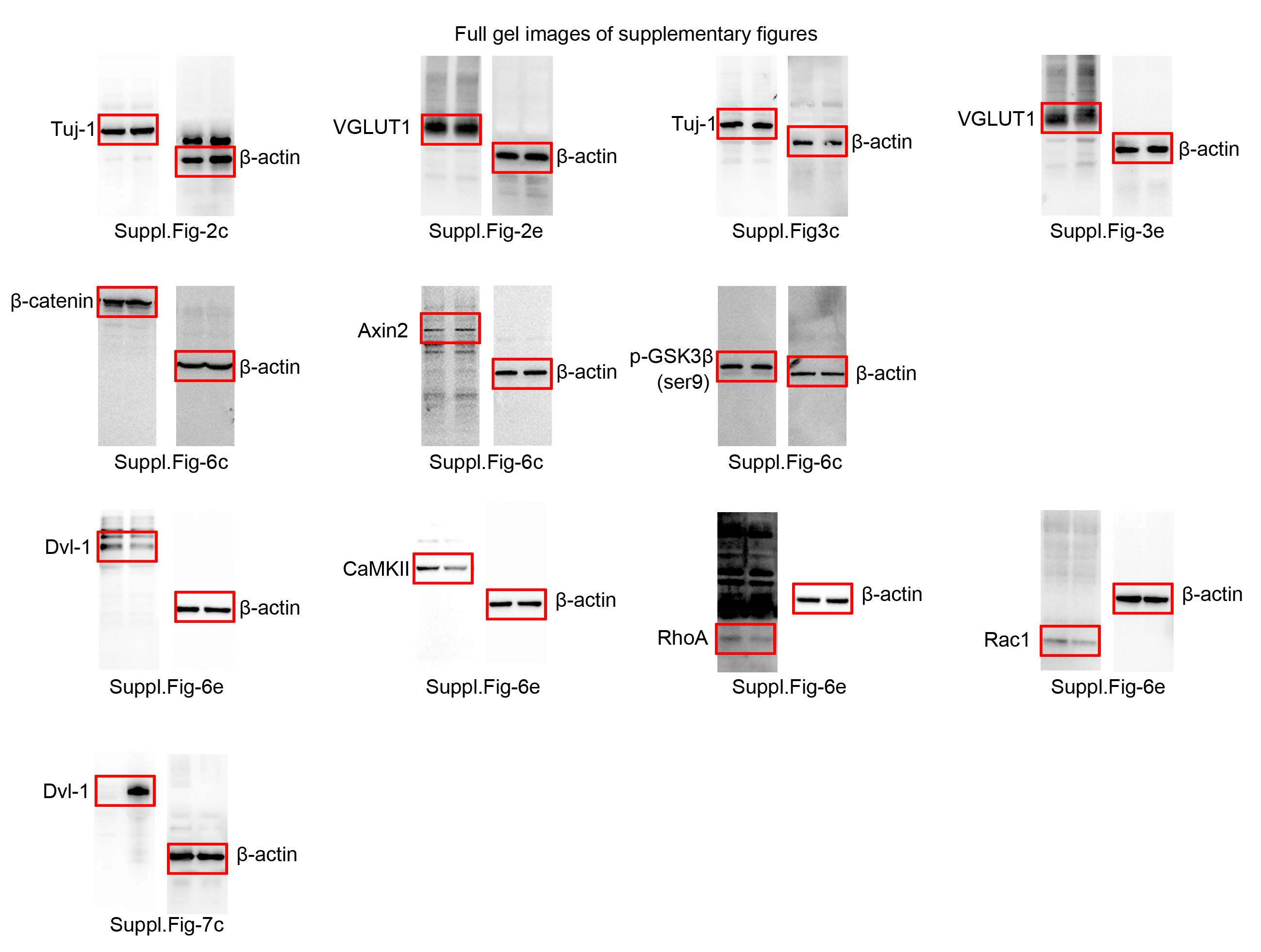

Supplement: Supplementary file 3 — Full gel images of supplementary figures. [file 12276_2025_1425_MOESM3_ESM.tif]
